# Insulin-like growth factor 2 as a driving force for exponential expansion and differentiation of the neonatal thymus

**DOI:** 10.1242/dev.204347

**Published:** 2025-04-10

**Authors:** Seung Woo Kang, Bryan R. Helm, Yu Wang, Shiyun Xiao, Wen Zhang, Anusha Vasudev, Ken S. Lau, Qi Liu, Ellen R. Richie, Laura P. Hale, Nancy R. Manley

**Affiliations:** ^1^ Department of Genetics, The University of Georgia, Athens, GA 30602, USA; ^2^ Department of Biostatistics, Vanderbilt University Medical Center, Nashville, TN 37232, USA; ^3^ Department of Epigenetics and Molecular Carcinogenesis, The University of Texas MD Anderson Cancer Center, Houston, TX 77030, USA; ^4^ Epithlielial Biology Center and Department of Cell and Developmental Biology, Vanderbilt University School of Medicine, Nashville, TN 37232, USA; ^5^ Department of Pathology and the Human Vaccine Institute, Duke University School of Medicine, Durham, NC 27710, USA

**Keywords:** Neonatal thymic expansion, *Foxn1*, Thymic total stroma, Thymic fibroblasts, IGF2, Human-mouse axis

## Abstract

Like all organs, the thymus grows in size and function rapidly during development, but this growth comes to a halt after birth. However, the molecular mechanisms behind such a transition in the thymus remain obscure. Using single-cell RNA sequencing (scRNA-seq) of the murine thymic stroma, we identified that major transcriptomic changes occur in the endothelium and mesenchyme across the transition to homeostasis. Differentially expressed gene and intercellular network analyses of temporally resolved scRNA-seq data revealed fibroblast-derived insulin-like growth factor 2 (IGF2) as a candidate driving neonatal thymic expansion. We demonstrated that IGF2 activity promotes a cortical thymic epithelial cell-specific proliferation and is tightly regulated at the thymic growth transition. Bulk RNA-seq of human thymi across the transition also revealed that IGF2 drives thymic expansion, suggesting an evolutionarily conserved role. Our study highlights the role of fibroblast-derived IGF2 in promoting cortical thymic epithelial cell proliferation and differentiation, resulting in early thymic expansion that is followed by downregulation to establish homeostasis.

## Teaser

**Summary:** IGF2 signaling to the thymic cortical epithelium drives a rapid expansion during the fetal and perinatal stages, while its downregulation causes a transition to thymic homeostasis in an evolutionarily conserved fashion.

## INTRODUCTION

The thymus is a primary lymphoid organ and a major source of self-restricted and self-tolerant naïve T-cells that shape the adaptive immunity. Neonatal humans and mice are immunocompromised partially due to unique T-cell production by the neonatal thymus. T-cells exported in neonates travel to an underpopulated periphery, resulting in an effector memory T-cell (T_EM_) phenotype (Adkins, 1991, 1999, 2003; Adkins and Hamilton, 1992, 1994; Opiela et al., 2009; Xiao et al., 2008). Neonatal T_EM_ cells are capable of rapidly responding to antigen stimulation but are unable to establish long-term memory (Akue et al., 2012; Zens et al., 2017), resembling virtual memory-like T-cells (T_VM_). This unique neonatal T_EM_ phenotype is likely shaped by a combination of neonatal hematopoietic intrinsic factors, underpopulated secondary lymphoid organs and a unique neonatal thymic microenvironment (Cuddihy et al., 2009; Gray et al., 2006; Srinivasan et al., 2021; Yang et al., 2015) that prevent development of a fully functional and diverse T-cell repertoire. However, neonatal T-cells alongside T_EM_ afig. stablend T_VM_ are suggested to play a unique role in the early response to infection (Kawabe et al., 2017; Li et al., 2005, 2007). Additionally, both murine and human data suggest the neonatal thymus plays a vital role in generating unique regulatory T (T_reg_) cells that confer life-long protection from autoimmunity (Mold et al., 2010; Yang et al., 2015), highlighting the importance of the neonatal thymic microenvironment in the proper development of the adaptive immune system.

The thymus, like other organs, must grow during fetal and postnatal development to keep pace with increasing body size, yet this expansion must halt and transition into homeostasis to maintain size and function (Lui and Baron, 2011). In murine thymus, such a transition occurs very early, at only 10 days after birth (P10) (Chen et al., 2009), but the mechanisms underlying this transition is not well understood. The cell cycle G1 checkpoint pathway cyclin D1-retinoblastoma (RB)-E2F axis regulates thymic growth (Garfin et al., 2013; Robles et al., 1996). Murine models with either null mutations in the RB family (Garfin et al., 2013) or with constitutive expression of Cyclin D1 under the control of a keratin 5 (K5) promotor (Robles et al., 1996) specifically in thymic epithelial cells (TECs) fail to undergo homeostasis. In these models, the thymus continues to grow with a concurrent increase in expression of *Foxn1*, a TEC-specific transcription factor essential for thymic organogenesis and postnatal function, which has E2F transcription factor-binding sites in its promoter that are regulated by RB. In the thymus, FOXN1 regulates both fetal and postnatal TEC differentiation and proliferation while maintaining postnatal thymic size and function (Chen et al., 2009; Manley and Condie, 2010; Manley et al., 2011; Nowell et al., 2011; Su et al., 2003; Vaidya et al., 2016). Despite its TEC-restricted expression in the thymus, *Foxn1* also indirectly affects development of the thymic vasculature and mesenchymal capsule in the non-TEC thymic stroma (Bryson et al., 2013; Mori et al., 2010; Nowell et al., 2011). *Foxn1* overexpression is sufficient to cause thymic overgrowth (Bredenkamp et al., 2014; Zook et al., 2011), suggesting regulation of the cyclin D1-RB-E2F-*Foxn1* axis is a molecular switch regulating the transition from neonatal expansion to juvenile homeostasis. However, how this switch is triggered to enable this transition is not understood.

In this study, we used time-series single-cell transcriptomic analyses to identify the molecular mechanism behind the transition from neonatal expansion to juvenile homeostasis in the perinatal murine thymus at postnatal day 10. To investigate the role of *Foxn1* in this transition, we used the *Foxn1^lacZ^* murine model, which exhibits normal thymic organogenesis and has comparable *Foxn1* levels to wild type until postnatal day 7, when *Foxn1* levels drop by ∼60% (Chen et al., 2009). Surprisingly, we found that the major changes in the thymic micro-environment across the thymic growth transition occurred similarly in both *Foxn1^+/+^* and *Foxn1^lacZ/lacZ^*. Using ligand enrichment analysis and intercellular signaling mapping, we identified IGF2 as a candidate factor in this transition. Additionally, our human bulk transcriptomic analysis also identified *IGF2* and its transcriptional regulator as the most differentially expressed genes across the thymic growth transition. IGF2 is well-categorized as a part of the imprinted gene network (IGN) and well-studied in cancer biology due to its highly mitogenic properties. However, its role in normal thymic development is not well studied. Here, we highlight the potential role of IGF2 in driving fetal and neonatal thymic growth prior to homeostasis both in mice and humans, with its downregulation as a crucial factor in the establishment of homeostasis.

## RESULTS

### Age- and *Foxn1*-dependent transcriptome patterns in the total thymic stroma

**Fig. 1.**
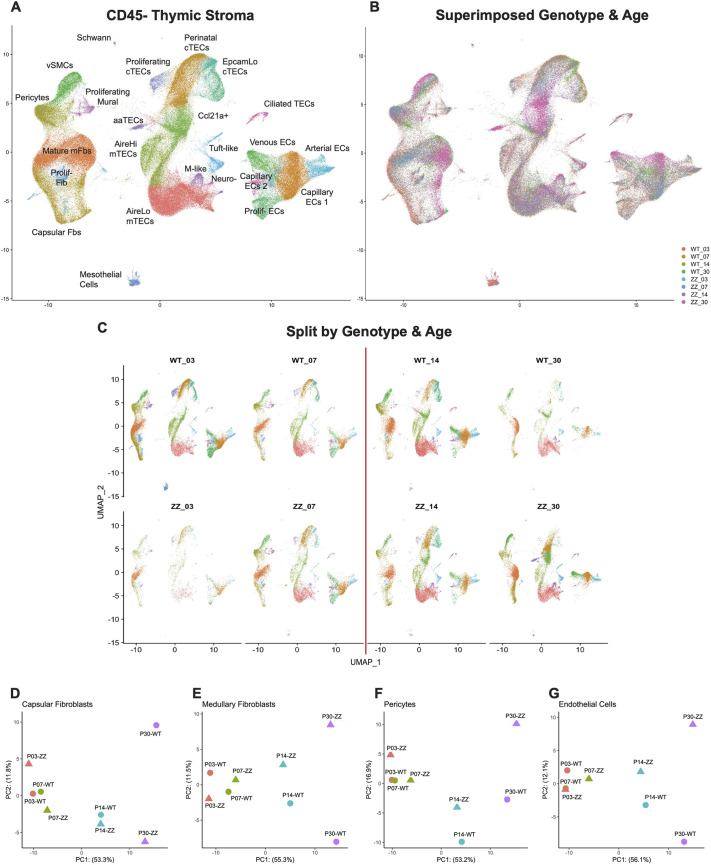
**scRNAseq of total thymic stroma from *Foxn1^lacZ^* shows dynamic stromal changes after the perinatal transition point.** (A) UMAP visualization of 206,271 CD45^−^ total thymic stromal cells with annotations. (B) UMAP visualization of superimposed datasets split by age and genotype. *Foxn1^lacZ^* wild-type littermates and *Foxn1^lacZ/lacZ^* homozygotes are denoted by WT and ZZ, respectively. Numbers indicate the age of the datasets. (C) UMAP visualizations of merged dataset split by age and genotype. The red line indicates postnatal day 10, a thymic transition point from neonatal expansion to juvenile homeostasis (the perinatal transition point). (D-G) PCA plots of pseudo-bulked data classes split by age and genotype.

Given the complex and mutually dependent interactions between TEC and other stromal cells types, we analyzed total stroma to capture this crosstalk, using both wild-type control mice and *Foxn1^lacZ/lacZ^* mice to identify *Foxn1-*dependent and -independent changes. First, we used time-series scRNA-seq to identify differences in the total thymic stroma of wild-type and *Foxn1^lacZ^* mice. We analyzed 3, 7, 14 and 30-day-old CD45^−^ total thymic stroma of males and females with multiple biological and technical replicates. Unsupervised clustering of 206,271 CD45^−^ total thymic stromal transcriptomes with Seurat (Butler et al., 2018; Hao et al., 2021; Satija et al., 2015; Stuart et al., 2019) revealed a complex non-hematopoietic thymic micro-environment. Using archetypal markers, we identified *Epcam*^+^, MHC-II^+^ thymic epithelial cells; *Pecam1*^+^, *Kdr*^+^, *Cldn5*^+^ endothelial cells; *Pdgfrb*^+^, *Acta2*^+^, *Myl9*^+^ vascular mural cells; *Pdgfra*+ fibroblasts; *Upk3b*^+^, *Lrrn4*^+^, *Nkain4*^+^ mesothelial cells (Huang et al., 2022); and *Gfap*^+^, *S100b*^+^ non-myelinating Schwann cells (Hu et al., 2018; Kousa et al., 2024) (Fig. 1A). Interestingly, we observed a rapid increase in the proportion of TECs between 3- and 7-day-old samples, which then hit a plateau (Fig. S1), suggesting TEC-focused thymic expansion in neonatal mice. While the proportion of fibroblasts decreased rapidly between 3- and 7-day-old samples, proportions of endothelium and pericytes remained similar across time (Fig. S1).

As an initial comparison, we superimposed the dataset separated by age and genotype to detect patterns (Fig. 1B). Thymic epithelial cells did not exhibit major differences across age and genotype, except for the appearances of previously described stress-responsive cortical TECs (cTECs) and aging-associated TECs (aaTECs) (Xiao et al., 2024 preprint) in 30-day-old male *Foxn1^lacZ/lacZ^*, located at the center of the UMAP (magenta-colored cell clusters) (Fig. 1B). Major transcriptome differences, visualized by non-overlapping colors, were observed in non-TEC stroma, especially capsular and medullary fibroblasts located at the bottom-left side and endothelial cells located at the bottom-right side of the UMAP (Fig. 1B). Regardless of *Foxn1* level, 3- and 7-day-old mesenchymal and endothelial cells clustered together, suggesting similar transcriptomic identities, while 14- and 30-day-old datasets clustered away from 3- and 7-day-old datasets (Fig. 1B). To take all the variables into consideration, we separated the merged dataset based on age and genotype with a red line indicating the thymic growth transition at postnatal day 10. With the exception of 30-day-old *Foxn1^lacZ/lacZ^*, every age-matched dataset exhibited a similar transcriptome regardless of *Foxn1* level (Fig. 1C).

To validate the major changes in endothelium and mesenchyme across the thymic growth transition point, we pseudo-bulked the age- and genotype-matched datasets, and generated PCA plots to compare the overall structure (Fig. 1D-G). PCA plots of fibroblasts, pericytes and endothelial cells unanimously showed age distribution across PC1 (*x*-axis), suggesting age mostly contributed to the variable transcriptomic identities, especially after the thymic transition to homeostasis. Postnatal day 3 and day 7 datasets exhibited overall similarities, whereas 14- and 30-day-old samples were unique (Fig. 1D-G). PC2 (*y*-axis) of the PCA plots revealed that decreased *Foxn1* affected the endothelium and mesenchyme as early as 14 days postnatally, but the largest effect on non-TEC stroma was seen in 30-day-old *Foxn1^lacZ/lacZ^* (Fig. 1D-G). Taken together, our data suggest a major non-TEC stromal change across the thymic growth transition is either upstream or independent of *Foxn1*. Although some *Foxn1*-dependent changes were observed in the total thymic stroma, these occurred after the thymic transition to homeostasis.

### Large shifts in the transcriptomic profile of thymic endothelium and mesenchyme across the thymic growth transition

**Fig. 2.**
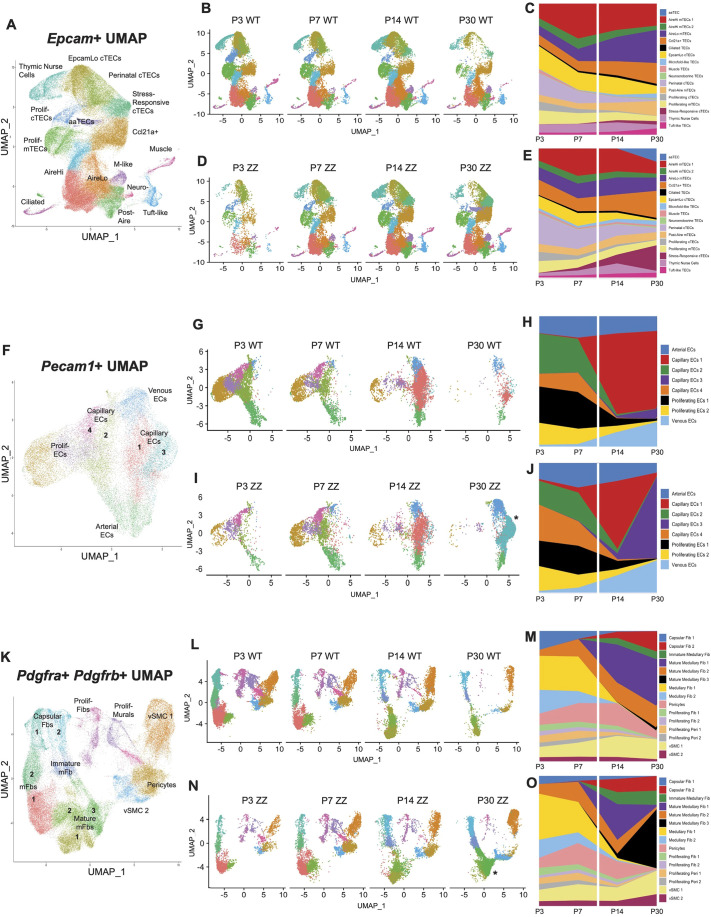
**Dynamic differences in thymic epithelial cells, endothelium and mesenchyme from subsampling of scRNAseq data.** (A,F,K) UMAP visualizations of thymic epithelial cells (TECs), endothelium and mesenchyme based on known markers with annotations. Cluster numbers were ranked based on the number of cells per cluster. (B,D,G,I,L,N) UMAP visualizations of subsampled datasets split by their age and genotype. (C,E,H,J,M,O) Percentage stacked charts showing the proportion of cell clusters across time. The white lines indicate the perinatal transition point. Asterisk in I and N indicates endothelium and mesenchyme clusters unique to P30 *Foxn1^lacZ/lacZ^* dataset.

To have a higher resolution on the transcriptomic changes before and after the thymic growth transition at postnatal day 10, we subsampled major stromal cell types: thymic epithelial cells, endothelial cells and mesenchymal cells. First, we subsampled *Epcam*^+^ TECs and identified various TEC populations based on their canonical markers: cTECs with varying degrees of *Epcam* (*Prss16*, *Syngr1*, *Ly75*, *Pax1* and *Tbata*), thymic nurse cells (*Prss16*, *Cd3e* and *Rag1*), medullary TECs (mTECs) with varying levels of *Aire* (*Aire*, *Cd52*, *Fezf2* and *Cd80*), proliferative cTECs and mTECs (*Top2a*, *Ccnd1* and *Mki67*), *Ccl21a*^+^ TECs (*Ccl21a*) (Baran-Gale et al., 2020), post-Aire TECs (*Spink5*), tuft-like TECs (*Avil* and *Pou2f3*), microfold-like TECs (*Gp2*, *Spib* and *Ccl20*), neuroendocrine TECs (*Snap25*, *Stxbp5l* and *Foxa3*), ciliated TECs (*Spag8*, *Dnah12* and *Dynlrb2*) and muscle TECs (*Myog, Myl1* and *Actc1*) (Michelson et al., 2022) alongside stress-responsive cTECs (*Atf3*, *Igfbp5*, *Gas1*, *Apoe*, *Fosb* and *Jun*) (Xiao et al., 2024 preprint) and aging-associated TECs (*Zeb2*, *Notch3* and *Prrx1*) (Kousa et al., 2024; Xiao et al., 2024 preprint) (Fig. 2A).

After separating the wild-type datasets based on age, we observed a decline in all of the cTEC populations over time, except for *Atf3*^+^ stress-responsive cTECs, which increased with age (Fig. 2B). Due to varying absolute number of cells per dataset class, we designed proportional graphs to visualize TEC subset change across time per genotype (Fig. 2C,E). In wild-type littermate samples, we observed a decrease in the perinatal cTEC population and an increase in the *Aire*^Lo^ mTEC population accompanied by a decrease in the *Aire*^Hi^ mTEC population across the thymic growth transition, denoted by a white line (Fig. 2C). Similar patterns could be observed from *Foxn1^lacZ/lacZ^* datasets (Fig. 2D,E). Interestingly, the proliferative cTEC population was drastically decreased after the transition to homeostasis and was not detectable in either 30-day-old *Foxn1^+/+^* or *Foxn1^lacZ/lacZ^*, whereas the proliferative mTEC population remained similar (Fig. 2C,E).

We then subsampled the *Pecam1*^+^ endothelial population to visualize endothelial cell (EC) changes across postnatal day 10. We determined four different endothelial subsets based on the conventional marker expression pattern: arterial (*Cxcl12*, *Gja4*, *Gja5* and *Edn3*), venous (*Vwf*, *Icam1* and *Vcam1*), capillary (*Car4*, *Kdr*, *Sgk1* and *Sparc*) (Kalucka et al., 2020) and proliferative population (*Top2a*, *Ccnd1* and *Mki67*) (Fig. 2F). We observed a loss of proliferative ECs (left side of the UMAP) with aging, whereas there was an increase in *Vwf*^+^ venous ECs with aging regardless of relative *Foxn1* level (Fig. 2G,I). Interestingly, we observed a major transcriptome shift in capillary and arterial ECs. Capillary ECs originally formed clusters 2 and 4 before forming new capillary EC clusters 1 and 3 across the thymic growth transition (Fig. 2G,H,I,J). Of note, new capillary EC cluster 3 was very prominent in 30-day-old *Foxn1^lacZ/lacZ^* (denoted by an asterisk in Fig. 2I) compared to the small proportion found in 30-day-old *Foxn1^+/+^* (Fig. 2I,J), which further supports that decreased *Foxn1* expression caused a late phenotype after the thymic growth transition.

Finally, we subsampled *Pdgfra*^+^ *Pdgfrb*^+^ mesenchymal populations comprising *Pdgfra*^+^ fibroblasts (Fbs) and *Pdgfrb*^+^ mural cells encompassing pericytes and vascular smooth muscle cells (vSMCs). Based on the quintessential markers, we identified capsular Fbs (*Pdgfra*, *Dpp4*, *Sfrp2* and *Lrrn4cl*), medullary Fbs (*Pdgfra* and *Serpine2*), mature medullary Fbs (*Pdgfra*, *Serpine2*, *Mmp9*, *Ltbp1* and *Col6a5*) (Nitta et al., 2021), vSMCs (*Pdgfrb*, *Myh11*, *Acta2* and *Myl9*) (Sorokin et al., 2020), pericytes (*Pdgfrb* and *Myl9*), and proliferative populations (*Top2a*, *Ccnd1* and *Mki67*) (Fig. 2K). Similar to cTECs and EC populations, proliferative mesenchyme subsets (middle of the UMAP) disappeared with age (Fig. 2L,N). Regardless of *Foxn1* level, both capsular fibroblasts (cFbs) and medullary fibroblasts (mFbs) formed different clusters after the transition to homeostasis, suggesting a major change in the transcriptome. For cFbs, cluster 1 disappeared and cluster 2 was formed after the transition to homeostasis. For mFbs, the appearance of mature mFbs clusters 1-3 accompanied the loss of mFbs clusters 1 and 2 across the thymic growth transition. Similar to the capillary ECs, new mature mFbs cluster 3 (denoted by an asterisk) was prominent in 30-day-old *Foxn1^lacZ/lacZ^* compared to a much smaller presence in 30-day-old *Foxn1^+/+^* (Fig. 2I,J,N,O), again suggesting a phenotype caused by decreased *Foxn1* expression after the transition to homeostasis. On the contrary, vSMCs and pericytes did not exhibit a major change after postnatal day 10 (Fig. 2L-O).

While major shifts in endothelial and mesenchymal populations were similar between WT and *Foxn1^lacZ/lacZ^* mutants, some differences were observed. Seven-day-old *Foxn1^lacZ/lacZ^* mutants demonstrated a premature appearance of *Atf3*^+^ stress-responsive cTECs (Xiao et al., 2024 preprint) that are absent in 7-day-old wild-type TECs (Fig. 2B-E). The relative proportion of these *Atf3*^+^ stress-responsive cTECs increased rapidly in *Foxn1^lacZ/lacZ^* (Fig. 2E), suggesting a unique cTEC subtype caused by a decreased *Foxn1* level in TECs. Similarly, in 30-day-old *Foxn1^lacZ/lacZ^* mutants, we observed a premature appearance of previously described aaTECs (Kousa et al., 2024; Xiao et al., 2024 preprint), characterized by a high epithelial-to-mesenchymal transition (EMT) signature (Fig. 2D,E). The proportion of the minority *Aire*^Lo^ mTEC subpopulation in 14- and 30-day-old *Foxn1^lacZ/lacZ^* was also substantially decreased compared to age-matched *Foxn1*^+/+^ mice (Fig. 2C,E). Additionally, gene signatures of capillary ECs, mature mFbs and vSMC2 unique to 30-day-old *Foxn1^lacZ/lacZ^* suggested a hypoxic and stressful thymic micro-environment (Fig. 2I,N), consistent with the appearance of *Atf3*^+^ stress-responsive cTECs and EMT aaTECs. As most of these changes occur well after the transitional window, they are likely indicators of the progressive effects of reduced *Foxn1* expression rather than of a role for *Foxn1* in the transition itself.

Overall, the most striking transcriptomic changes occurred in the thymic endothelium and mesenchyme across the thymic growth transition at postnatal day 10, which were *Foxn1* independent. *Foxn1*-dependent transcriptomic changes in the thymic endothelium and mesenchyme were observed but occurred before or after the transition. Combined, these data suggest that a signal acting upstream of *Foxn1* is driving the thymic transition from neonatal expansion to juvenile homeostasis.

### IGF2 is a potential switch that regulates neonatal expansion to juvenile homeostasis in murine thymus

To further investigate this potential signal, we utilized CellChat (Jin et al., 2021) to detect changes in intercellular signals before and after the thymic growth transition. Since the ligand-receptor pair database is utilized to build intercellular networks, we used the ligand enrichment analysis to compare total thymic stroma from 7-day-old and 14-day-old *Foxn1^+/+^* samples, and separately compared 7-day-old and 14-day-old *Foxn1^lacZ/lacZ^* samples.

**Fig. 3.**
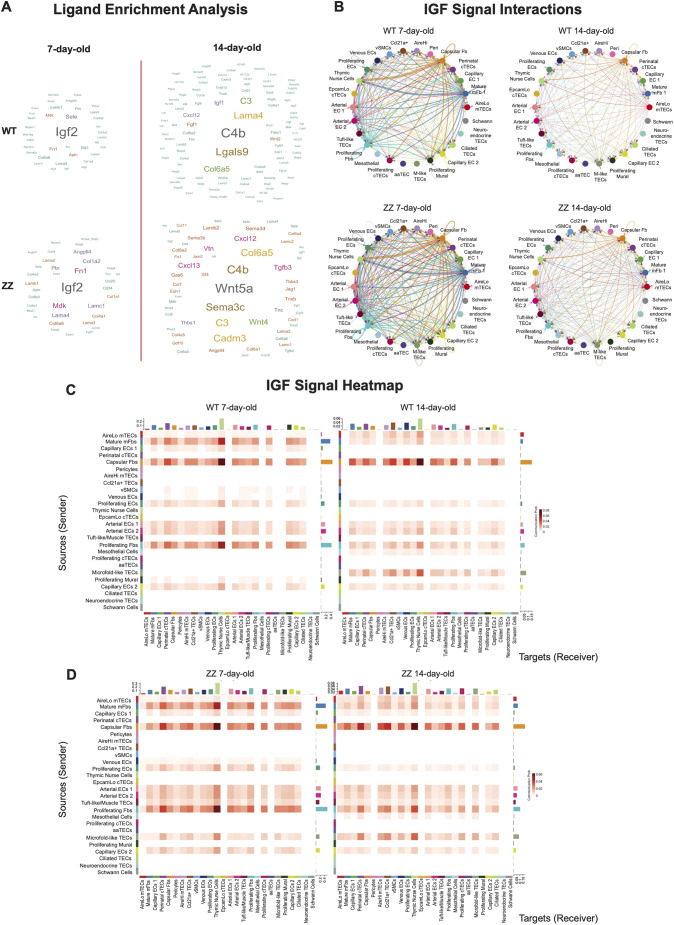
**IGF2 and IGF signaling is increased during neonatal expansion of the thymus.** (A) Ligand enrichment comparison of 7-day-old and 14-day-old *Foxn1^+/+^* (top panels) and 7-day-old and 14-day-old *Foxn1^lacZ/lacZ^* (bottom panels). The red line indicates the perinatal transition point. (B) Circle plots of IGF interactions (*Igf2*-*Igf1r*) among different cell types. The direction and thickness of arrows indicate interaction strengths that are deduced from the weight of ligand and/or receptor expression on a per cell basis. The top panels are *Foxn1^+/+^*, bottom panels are *Foxn1^lacZ/lacZ^*. (C,D) Heat maps of IGF interactions (*Igf2*-*Igf1r*) among different cell types. The *y*-axis indicates the clusters expressing ligands; the *x*-axis indicates the clusters expressing receptors.

The predominant signal in 7-day-old *Foxn1^+/+^* was *Igf2*, whereas *C4b*, *C3*, *Wnt2*, *Fgf1* and *Cxcl12* were highly expressed in 14-day-old *Foxn1^+/+^*, while *Igf2* was absent (Fig. 3A). The comparison of 7- and 14-day-old *Foxn1^lacZ/lacZ^* datasets also identified *Igf2* as the major signal at 7 days, which was also absent at 14 days, suggesting that this downregulation was independent of *Foxn1* expression levels and could be acting upstream of *Foxn1.* After the transition, however, the signaling landscape differed from that seen in wild-type samples, with *Wnt5a*, *Wnt4*, *Tgfb3*, *Jag1*, *Cxcl12*, *Cxcl13* and *Gas6* enriched in 14-day-old *Foxn1^lacZ/lacZ^*, and *C4b*, *C3*, *Wnt2*, *Fgf1* and *Cxcl12* were highly expressed in 14-day-old *Foxn1^+/+^*. A pseudo-bulked differentially expressed gene (DEG) analysis using a logistic regression test also revealed *Igf2* as one of the most differentially expressed gene across the thymic growth transition (Table 1). Additionally, we compared 3- and 7-day-old thymi to better understand intercellular interactions during the perinatal period. Comparison of 3- and 7-day-old *Foxn1^+/+^* yielded similar intercellular signal profile, including IGF signal (Fig. S2A,C), which was also present in 3- and 7-day-old *Foxn1^lacZ/lacZ^* thymi (Fig. S2B,D). Interestingly, comparison of 3- and 7-day-old *Foxn1^lacZ/lacZ^* also showed WNT signaling between these time points, indicating that the WNT signal seen at 14-day-old *Foxn1^lacZ/lacZ^* increased before the transition.

**Table 1.** Top 5 DEGs of pseudo-bulked endothelium and mesenchyme

Gene	*P*-value	avg_log2FC	pct.1	pct.2	*P*_val_adj
Endothelium					
*Mest*	0	1.915944891	0.811	0.251	0
*Ccnd1*	0	1.208513309	0.872	0.532	0
*Igf2*	0	1.081733948	0.55	0.146	0
*Apln*	0	0.986626533	0.446	0.089	0
*Rbm3*	0	0.982894688	0.909	0.639	0
Mesenchyme					
*Dlk1*	0	3.036904601	0.836	0.121	0
*Mest*	0	2.244602716	0.844	0.204	0
*Igf2*	0	2.166934013	0.9	0.273	0
*Peg3*	0	2.162785689	0.882	0.383	0
*Itm2a*	0	2.154728963	0.914	0.569	0

Strikingly, circle plots visualizing IGF signal comparison between 7- and 14-day-old datasets revealed IGF2-IGF1R interaction strengths were decreased in 14-day-old thymi regardless of *Foxn1* level (Fig. 3B). In both 7-day-old *Foxn1^+/+^* and *Foxn1^lacZ/lacZ^*, capsular, mature medullary and proliferative fibroblasts sent strong IGF signals to other stromal cells in both autocrine and paracrine fashion. In contrast, in 14-day-old *Foxn1^+/+^* and *Foxn1^lacZ/lacZ^*, capsular fibroblasts were the sole source of IGF ligand and had substantially reduced overall relative signal strength (Fig. 3B). Heatmap analysis of interaction probability of an IGF signal between IGF2 and IGF1R further supported these observations (Fig. 3C). In 7-day-old *Foxn1^+/+^* and *Foxn1^lacZ/lacZ^*, capsular, mature medullary and proliferating fibroblasts sent IGF signals to thymic nurse cells, perinatal cTECs, *Aire*^Hi^ mTECs and *Ccl21a*^+^ TECs (Fig. 3C,D). In 14-day-old *Foxn1^+/+^* and *Foxn1^lacZ/lacZ^*, only capsular fibroblasts were a source of IGF signal and its strength was significantly reduced (Fig. 3C,D). Of note, 14-day-old *Foxn1^+/+^* and *Foxn1^lacZ/lacZ^* comparisons showed wild type expressing higher levels of *Ccl25* (Fig. S3A), a direct *Foxn1*-downstream target expressed on cTECs that is essential for thymopoiesis. Total intercellular interactions were reduced in 14-day-old *Foxn1^lacZ/lacZ^* compared to *Foxn1*^+/+^, particularly signals received by perinatal cTECs (Fig. S3B). Together, our intercellular network analyses strongly suggest that IGF2-mediated proliferation drives the exponential growth and expansion of the neonatal murine thymus, and acts upstream of *Foxn1*.

### Dynamic IGF2 and IGF-related gene expression in total thymic stroma and TECs across the thymic growth transition

**Fig. 4.**
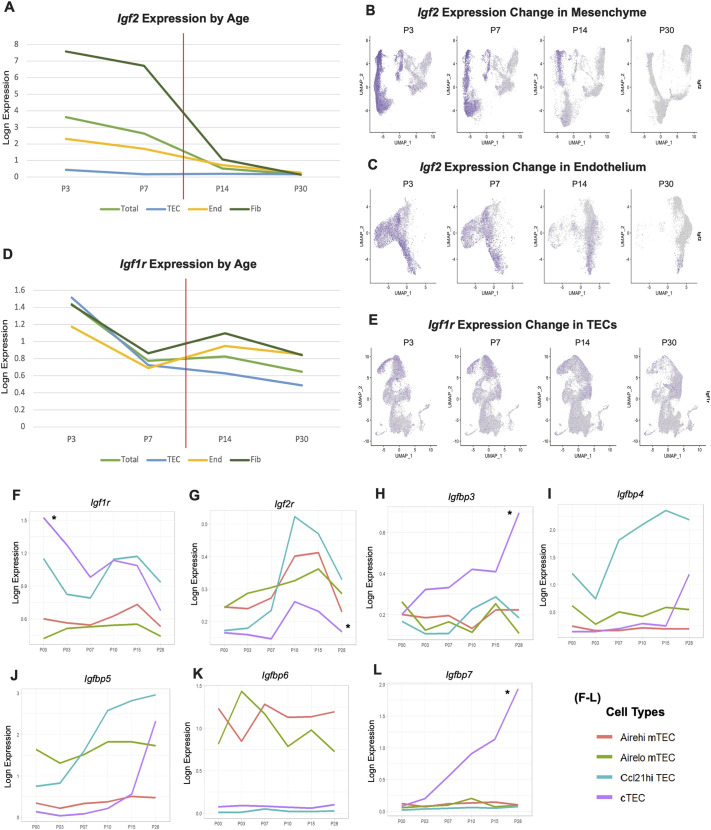
***Igf2* and *Igf1r*-related gene expression varies with aging.** (A,D) Average expression of *Igf2* and *Igf1r* calculated from total and subsampled scRNAseq across time. The red lines indicate the perinatal transition point. (B,C) Feature plot visualization of *Igf2* in *Pdgfra*^+^ *Pdgfrb*^+^ mesenchyme (B) and *Pecam1*^+^ endothelium (C) across time. (E) Feature plot visualization of *Igf1r* in *Epcam*^+^ thymic epithelial cells (TECs) across time. (F-L) Data were acquired from a new time-series scRNAseq of TECs from 0-, 3-, 7-, 10-, 15- and 28-day-old thymi. Line graphs show the average expression of IGF2 receptors and IGF-binding proteins across time in different TEC subsets. End, endothelial cells; Fib, fibroblasts. Asterisks are added for visualization purposes.

IGF2 mediates murine embryonic growth during development through interactions with IGF1R that promote cell proliferation and prevent apoptosis. In contrast, IGF2R acts to inhibit the pathway by targeting IGF2 for lysosomal degradation (Chao and D'Amore, 2008; Plasschaert and Bartolomei, 2014; Sélénou et al., 2022). IGF2-IGF1R interaction promotes cyclin D1 transcription (Hartmann et al., 2005; Preusser et al., 2018), which hyper-phosphorylates RB and enables E2F-mediated transcriptions (Harbour and Dean, 2000; Kim et al., 2022; Narasimha et al., 2014). As cyclin D1-RB-E2F-mediated regulation of *Foxn1* expression is a crucial regulator of the transition to homeostasis (Garfin et al., 2013), its downregulation upstream of *Foxn1* is even more significant. To understand how *Igf2* and *Igf1r* expression changes over time, we calculated their average expression in the P3, P7, P14 and P30 scRNAseq datasets and plotted them as line graphs (Fig. 4A,D). We analyzed the expression of *Igf2* and *Igf1r* in TECs, endothelium and fibroblasts, as well as the total average expression at each time point. *Igf2* was highly expressed in fibroblasts prior to the thymic growth transition, and from P7 to P14 its expression in fibroblasts decreased by ∼85%. *Igf2* expression in endothelial cells showed a more gradual decline over time (Fig. 4A). *Igf2* expression in all of the subsets was not detectable at P30 (Fig. 4A).

In the mesenchyme subsampled dataset, we observed high levels of *Igf2* expression in capsular, medullary and proliferative fibroblasts in 3- and 7-day-old datasets, which disappeared after postnatal day 10, except for a capsular fibroblast subset in 14-day-old datasets (Figs 4B, 2K). In the endothelium subsampled dataset, we observed high levels of *Igf2* expression in arterial, capillary and proliferative endothelial cells in 3- and 7-day-old datasets. *Igf2* expression remained detectable in arterial ECs of 30-day-old datasets (Figs 4C, 2F), suggesting a potential role in adult EC proliferation. *Igf1r* expression was decreased by ∼50% in all of the major stroma at postnatal day 7, immediately before transition to homeostasis. At P14, *Igf1r* expression level in endothelium and fibroblasts was increased compared to P7, suggesting enhanced *Igf1r* during the thymic growth transition (Fig. 4D). However, cTEC and *Ccl21a*^+^ TEC-restricted *Igf1r* expression in TECs continued to decrease during this time (Fig. 4D,E).

We analyzed a separately generated TEC-sorted wild-type time-series scRNAseq dataset to acquire a higher resolution of IGF-associated gene changes in TECs. *Igf1r* was highly expressed in cTECs (denoted by an asterisk, Fig. 4F) and *Ccl21a*+ TECs compared to mTECs (Fig. 4F), supporting our previous observations. In cTECs, *Igf1r* expression rapidly decreased until postnatal day 7 and transiently increased at postnatal day 10, after which it decreased again (Fig. 4F). In contrast, IGF2-inhibitory *Igf2r* expression in cTECs, *Ccl21a*+ TECs and *Aire*^Hi^ mTECs increased at postnatal day 10 (Fig. 4G), suggesting tightly regulated IGF2 activity at the thymic growth transition to homeostasis.

IGF-binding proteins (IGFBPs) bind to IGF1 and IGF2 to regulate their activities in a context-specific fashion (Allard and Duan, 2018). We surveyed the expression of murine IGFBP family genes (*Igfbp1*-*Igfbp7*) in our TEC scRNAseq dataset (Fig. 4H-L, Fig. S4A,B). Anti-proliferative *Igfbp3* and *Igfbp7* expression (Jin et al., 2020) rapidly increased in cTECs over time (Fig. 4H,L), supporting a selective decrease in IGF signaling in cTEC with age. Interestingly, IGF2-specific inhibitor *Igfbp6* (Bach et al., 2013) (Fig. 4K) was exclusively expressed in mTEC subsets at all ages examined, suggesting IGF2 activity is actively inhibited in the thymic medulla. Additionally, expression of both *Igfbp4* and *Igfbp5* that induce cellular senescence (Sanada et al., 2018) were increased in *Ccl21a*^+^ TECs across the perinatal stage (Fig. 4I,J), further supporting IGF2 inhibition in mTECs. Both *Igfbp4* and *Igfbp5* expression in cTECs were also increased after postnatal day 15 (Fig. 4I,J), consistent with inhibition of IGF2 signaling. *Igfbp1* was exclusively detected in mTECs, but the overall expression was extremely low (Fig. S4A). In addition, *Igfbp2* was highly expressed in neonatal cTECs and *Ccl21a*^+^ TECs, which immediately dropped at postnatal day 3 and maintained a plateau until it increased in cTECs at postnatal day 28 (Fig. S4B). Overall, our two independent time-series scRNAseq datasets strongly suggest that IGF2 signaling in cTECs is driving perinatal exponential thymic expansion, and IGF2 inhibition causes the transition to thymic homeostasis.

### Capsular IGF2^+^ fibroblasts may drive neonatal thymic expansion prior to homeostatic transition

**Fig. 5.**
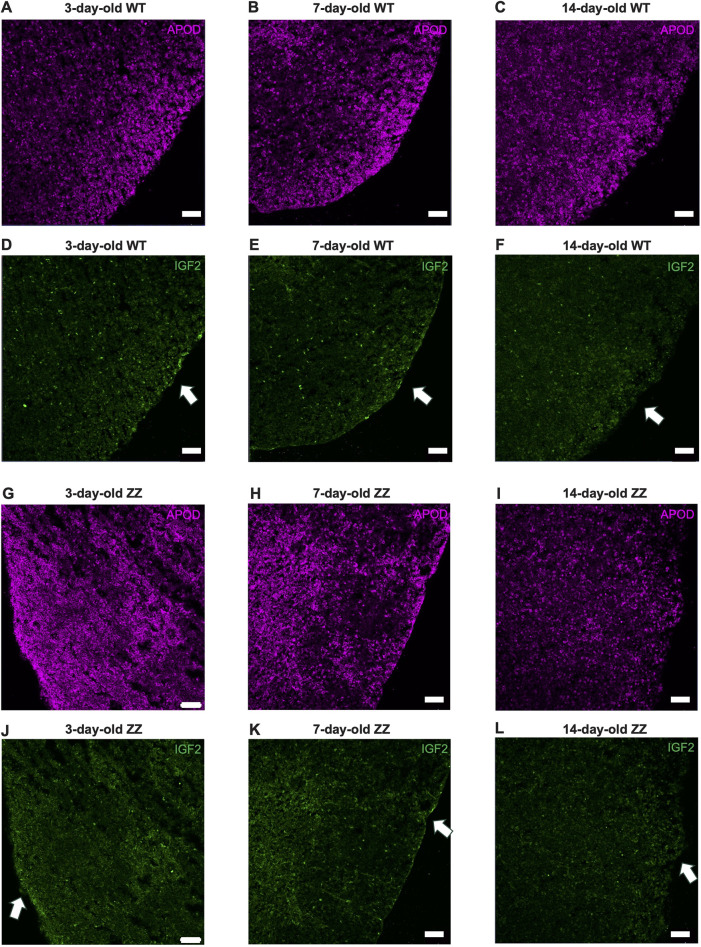
**Prominent capsular IGF2 expression during murine neonatal thymic expansion.** (A-L) Immunohistochemistry of frozen thymic sections (10 µm). (A-C) APOD^+^ fibroblasts in 3-, 7- and 14-day-old *Foxn1^+/+^* males. (D-F) IGF2 distribution in the identical sections to A-C. Arrows indicate areas of high APOD concentration. (G-I) APOD^+^ fibroblasts in 3-, 7- and 14-day-old *Foxn1^lacZ/lacZ^* males. (J-L) IGF2 distribution of the identical sections to G-I. Arrows indicate areas of high APOD concentration. Wild type (WT), *Foxn1^+/+^*; ZZ, *Foxn1^lacZ/lacZ^*. *n*=3 per category. Scale bars: 50 µm.

Our scRNAseq data suggested mesenchyme, especially fibroblasts, is the main source of *Igf2* that could mediate the proliferation of cTECs. To validate our scRNAseq data, we used immunohistochemistry (IHC) to acquire *in situ* distribution of mesenchyme and soluble IGF2 protein. Due to CD3^+^ thymocytes expressing IGF receptors (Fig. S5A,B), measuring the proximity between IGF2 and IGF1R on TEC with IHC was not feasible. Age- and genotype-matched thymi to scRNAseq datasets were collected and processed for frozen IHC (Fig. 5, Figs S6-S8). We chose apolipoprotein D (APOD) as a fibroblast marker, identified from our scRNAseq data. There was an overall thinning of capsular fibroblasts across these time points, consistent with thymic expansion, and capsular fibroblasts were not prominent in 14-day-old thymic sections (Fig. 5A-C). IHC with podoplanin also showed strong capsular fibroblasts in 3-day-old thymi, which decreased with age (Fig. S9). Strong IGF2 protein was observed throughout the subcapsular area in 3- and 7-day-old *Foxn1^+/+^* thymi, whereas capsular IGF2 was not detected in 14-day-old *Foxn1^+/+^* (Fig. 5D-F, Fig. S7A-C). IGF2 signal coincided with APOD^+^ fibroblast levels, further suggesting capsular fibroblasts were the source of IGF2 (Fig. 5A-F).

Similar to *Foxn1^+/+^*, a decrease in capsular fibroblast distribution was observed with age in *Foxn1^lacZ/lacZ^* thymi, (Fig. 5G-I). There was also a decrease in fibroblast frequency in 14-day-old *Foxn1^lacZ/lacZ^* thymi compared to 3- and 7-day-old thymi (Fig. 5I). As in *Foxn1^+/+^* thymi, IGF2 signal correlated with APOD^+^ fibroblast detection. There were strong IGF2 signals in the capsule of 3- and 7-day-old *Foxn1^lacZ/lacZ^* thymi, whereas IGF2 was not detected in 14-day-old *Foxn1^lacZ/lacZ^* (Fig. 5J-L, Fig. S7D-F). Additionally, we observed an overall greater IGF2 signal in the thymic cortex with high IGF2 signal with vasculature, likely from endothelial cells and pericytes (Fig. 5D-F,J-L, Fig. S7A-F). Moreover, there were high levels of IGF2 in the cortico-medullary junction (CMJ) and medulla in 3- and 7-day-old *Foxn1^+/+^* and *Foxn1^lacZ/lacZ^* thymi (Fig. S7G,H,J,K). Interestingly, we detected IGF2 in the 14-day-old *Foxn1^+/+^* CMJ and medulla (Fig. S7I), but not in the 14-day-old *Foxn1^lacZ/lacZ^* (Fig. S7I,L). Altogether, our IHC analyses strongly suggest capsular IGF2 from capsular fibroblasts plays a role in driving exponential thymic expansion before postnatal day 10.

### TEC-specific deletion of IGF1R impairs early thymopoiesis

**Fig. 6.**
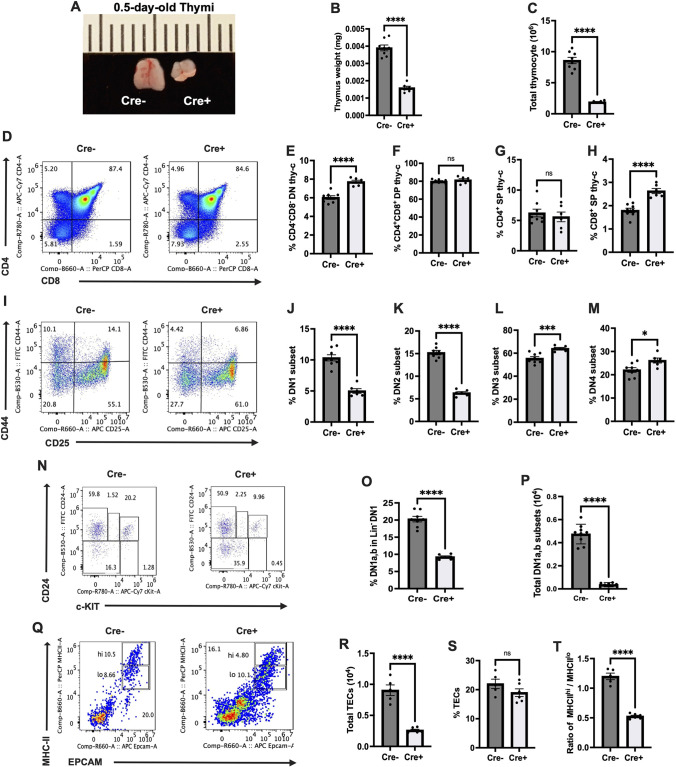
**Thymic epithelial cell-specific IGF1R deletion results in impaired thymopoiesis.** (A) Thymic size comparison between *Foxn1*^+/+^×Igf1r^tm2Arge/tm2Arge^ (Cre^−^) and *Foxn1*^Cre/+^×Igf1r^tm2Arge/tm2Arge^ (Cre^+^). Scale is in mm. (B,C) Thymic weight and thymocyte number comparison between Cre^−^ and Cre^+^. (D-H) Representative image (D) and percentage (E-H) of CD4^−^CD8^−^ DN, CD4^+^CD8^+^ DP, CD4^+^ SP and CD8^+^ SP thymocytes, separated by genotype. (I-M) Representative image (I) and percentage (J-M) of CD25^−^CD44^+^ DN1, CD25^+^CD44^+^ DN2, CD25^+^CD44^−^ DN3 and CD25^−^CD44^−^ DN4 thymocytes, separated by genotype. (N) DN1a,b gating strategy. CD24^+^ cKit^Hi^ cells were selected from Lin^−^ DN1 (Lin^−^ CD44^+^ CD25^−^) cells. (O,P) Relative DN1a,b percentage and total number of DN1a,b, separated by genotype. (Q-T) Representative image (Q) and number and percentage (R-T) and percentage of thymic epithelial cells (TECs), and ratio of MHCII^Hi^ TECs to MHCII^Lo^ TECs, separated by genotype. Each dot represents a result from one experiment. Data are mean±s.e.m. *P<0.05, ***P<0.001, ****P<0.0001 (unpaired Student's *t*-test).

To further test the role of fibroblast-derived IGF2 on TECs, *Igf1r* was specifically deleted in TECs by crossing *Foxn1*-Cre to *Igf1r*^tm2Arge/tm2Arge^. TEC-specific deletion of *Igf1r* resulted in significantly smaller neonatal thymi, an approximately 62.5% decrease in weight (Fig. 6A,B), with a similar reduction in total thymocyte number (Fig. 6C). Absolute numbers of CD4^−^CD8^−^ double-negative 1-4 (DN1-4), CD4^+^CD8^+^ double-positive (DP), CD4^+^ single-positive (SP) and CD8^+^ SP thymocytes were all significantly decreased (Fig. S10A-H). However, relative proportions of thymocyte subsets showed altered thymopoiesis in neonatal mice lacking *Igf1r* in TECs, compared to controls. Proportions of CD4^−^CD8^−^ DN and CD8^+^ SP thymocytes were significantly increased in neonatal mutants, whereas proportions of DP and CD4^+^ SP thymocytes remained unchanged (Fig. 6D-H). Further analysis of CD4^−^CD8^−^ DN thymocytes revealed significantly decreased proportions of CD25^−^CD44^+^ DN1 and CD25^+^CD44^+^ DN2 thymocytes in neonatal Cre^+^ mice, accompanied by increased proportions of CD25^+^CD44^−^ DN3 and CD25^−^CD44^−^ DN4 thymocytes (Fig. 6I-M), suggesting impaired T-lineage specification. Analysis of cKit^Hi^ early thymic progenitor (ETP) cells revealed significantly reduced number and proportion of (alpha beta) early intrathymic T-cell progenitor cells (DN1a,b) within Lin^−^ DN1 (Fig. 6N-P), further supporting impaired T-lineage specification in neonatal Cre^+^ mutants. TEC-specific deletion of IGF1R significantly reduced the number of TECs, while the proportion of TECs remained unchanged (Fig. 6Q-S), but with a significant decrease in the MHCII^Hi^ TEC to MHCII^Lo^ TEC ratio (Fig. 6T). Overall, these results strongly support a unique role of IGF signaling specifically in TECs, with phenotypes that are consistent with known roles for *Foxn1*.

### IGF2 may drive human neonatal thymic expansion

**Fig. 7.**
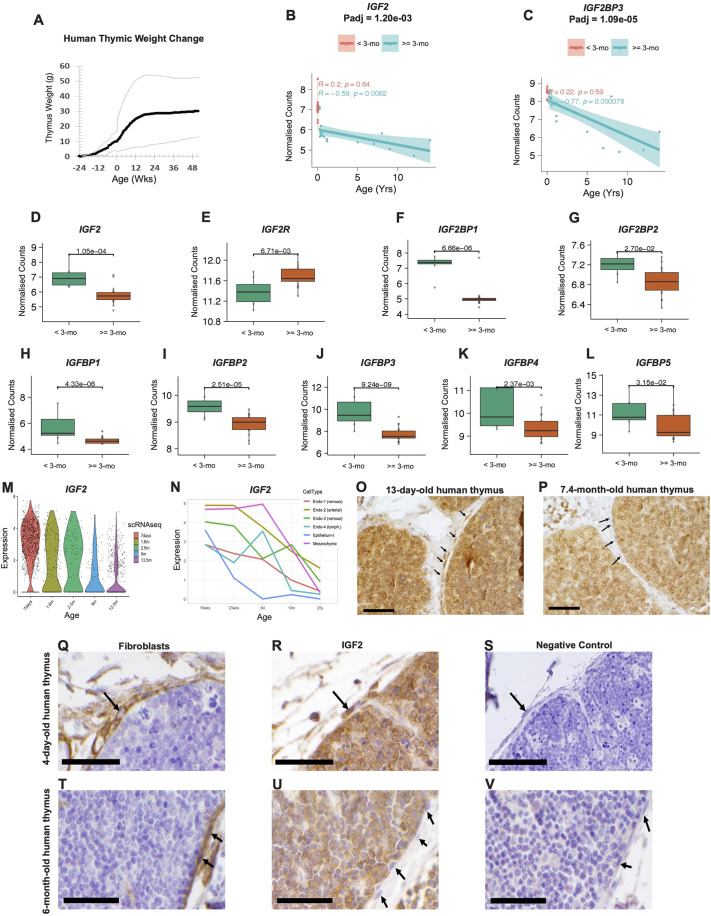
**IGF2 expression is increased during human neonatal thymic expansion.** (A) Human thymus weights compiled from published sources (Archie et al., 2006; Pryce et al., 2014) were replotted as a function of age. Birth is defined as day/week 0. The 5th and 95th percentiles are indicated by grey lines. (B,C) Scatter plot visualizations showing *IGF2* and *IGF2BP3* across age. Pink indicates samples before 3 months, teal indicates samples after 3 months. (D-L) Box and whisker plots visualizing IGF2-related genes before and after 3 months (0.25 years). The box represents the interquartile range (IQR), with the line inside showing the median, and the whiskers extend to the most extreme data points within 1.5 times the IQR, while data points beyond the whiskers are considered outliers. (M) Violin plot visualizing *IGF2* expression in thymic fibroblasts, using published human thymus scRNAseq dataset (Heimli et al., 2022). R, correlation coefficient; *P*, adjusted *P*-values calculated using a Wald test. (N) Line graph visualizing IGF2 expression in the thymic stroma, using published human thymus scRNAseq dataset (Bautista et al., 2021). (O-V) Immunohistochemistry of formalin-fixed, paraffin wax-embedded human thymic tissues. Scale bars: 50 µm. (O,P) Subcapsular cortex strongly reacts with IGF2 monoclonal antibody (mAb; brown) in thymus from a 13-day-old human infant (O), which is minimal to absent in a 7.4-month-old human infant (P). Arrows indicate fibroblasts. (Q-V) Fibroblasts (arrow in Q) overlying subcapsular cortex strongly react with TE7 mAb (brown) in thymus from both a 4-day-old human infant (Q) and a 6-month-old infant (T). These fibroblasts react strongly with IGF2 in the 4-day-old thymus (R, arrow) but such reactivity is minimal to absent in thymus from the 6-month-old infant (U, arrows). No reaction is seen when these tissues are incubated with mouse IgG as a negative control (S,V). Arrows in S and V indicate fibroblasts.

We mapped human thymic weight over time, using data obtained from autopsy of 3777 female and male subjects ranging from 16 weeks gestational age (shown as −24 weeks; birth as 0 weeks) to 52 weeks after birth (Archie et al., 2006; Pryce et al., 2014). In humans, the thymic transition from growth to homeostasis occurred between 12 and 16 weeks after birth (Fig. 7A). We then performed bulk RNA sequencing with 28 perinatal human thymi (Table 2) and stratified the datasets into two groups based on age: before and after 3 months.

**Table 2.** Human thymus sample demographic

Sample ID	Age (years)	Sex
T2010	0.5	Male
T2012	0.008	Male
T2013	8	Male
T2015	0.25	Female
T2016	0.013	Female
T2018	0.3	Female
T2024	0.416	Female
T2025	0.25	Female
T2027	5	Female
T2033	9	Female
T2035	1.167	Female
T2036	7	Female
T2038	12	Male
T2039	14	Female
T2044	1.166	Female
T2046	0.008	Male
T2048	0.008	Female
T2050	0.328	Male
T2052	0.021	Male
T2055	0.328	Male
T2056	0.008	Male
T2057	0.024	Female
T2059	0.833	Male
T2060	0.583	Male
T2063	0.333	Female
T2064	0.25	Male
T2065	0.4166	Female
T2067	0.016	Female

DEG analysis between the two groups yielded *IGF2* and *IGF2BP1* as the most differentially expressed genes. Mapping *IGF2* expression over time and separating into two groups before and after 3 months (0.25 years) showed a striking pattern. *IGF2* expression is highly maintained until 3 months, when there is an immediate drop in expression after the transition to homeostasis, with a strong negative correlation as aging continues (Fig. 7B). Transcription of IGF2BP3, promoting stability and translation of IGF2 (Nielsen et al., 2003), exhibited a similar pattern with age, though the decrease in expression across the thymic growth transition was not as dramatic compared to *IGF2* (Fig. 7C). Overall, *IGF2* and its translational regulators were significantly decreased after the transition to homeostasis (Fig. 7D,F,G), suggesting an identical role of *IGF2* mediating neonatal thymic expansion in human thymus.

In contrast to the mouse data, the expression of *IGF2* receptors *IGF1R* and *INSR* did not change with age (Table 3). However, the expression of the inhibitory *IGF2R* increased after the transition to homeostasis (Fig. 7E), consistent with the results in mice. Expression of IGFBP family proteins (*IGFBP1*-*IGFBP5*) was significantly reduced after 3 months (Fig. 7H-L). Our reanalysis of published scRNAseq datasets (Bautista et al., 2021; Heimli et al., 2022) revealed patterns in human thymic fibroblasts similar to those in mice, with a substantial decrease in *IGF2* expression in human thymic mesenchyme and/or fibroblasts after 3 months (Fig. 7M,N), further supporting our hypothesis. Our IHC further validated this finding, showing strong IGF2 in the 13-day-old human thymic subcapsular area (Fig. 7O) that was considerably reduced in 7.4-month-old human thymic tissue (Fig. 7P). Higher magnification of 4-day-old and 6-month-old human thymic subcapsular area also showed strong IGF2 staining in thymic capsular fibroblasts of 4-day-old thymus, which is absent in 6-month-old thymus (Fig. 7Q-V). Combined, our human data strongly suggest that IGF2 also drives exponential expansion of the neonatal human thymus, and that its downregulation could trigger the transition to homeostasis.

**Table 3.** Age-independent expression of *IGF1*, *IGF1R* and *INSR* in human thymus

Gene	Mean	log2FoldChange	lfcSE	stat	*P*-value	*P*adj	Weight
*IGF1*	9.934	−0.611314943	0.311	−1.963	0.0496	0.158	0.844
*IGF1R*	1238.4	−0.223700525	0.097	−2.299	0.0215	0.064	1.332
*INSR*	416.85	−0.292287167	0.135	−2.159	0.0308	0.097	1.058

## DISCUSSION

Thymic size and function rapidly increase until 10 days (mice) (Chen et al., 2009) and 3 months (humans) (Fig. 7A) after birth, after which they reach a plateau and maintain homeostasis. However, the molecular mechanism behind this transition remains unclear. The transcription factor *Foxn1* is a lynchpin of TEC biology, as it determines TEC differentiation, proliferation and maintenance, as well as the development of non-TEC thymic stroma (Bryson et al., 2013; Chen et al., 2009; Manley and Condie, 2010; Mori et al., 2010; Nowell et al., 2011; Su et al., 2003; Vaidya et al., 2016). Here, using time-series single-cell RNA sequencing of *Foxn1*-hypomorphic model *Foxn1^lacZ^*, we investigated the molecular mechanism underlying the thymic growth transition as well as the potential role of *Foxn1*. *Foxn1^lacZ^* mice have an approximately 60% decrease in *Foxn1* expression beginning at postnatal day 7 (Chen et al., 2009). Both the early high levels of IGF2 signaling and its downregulation at the transition were unaffected in *Foxn1^lacZ^* mutants, suggesting that these mechanisms regulating the transition from neonatal expansion to juvenile homeostasis act upstream of *Foxn1*. This conclusion is also consistent with the known ability of IGF2 signaling to act through the cyclin D1-RB-E2F pathway, which also regulates *Foxn1* expression in TECs, making IGF2 a clear candidate to trigger this transition to homeostasis.

Decreased *Foxn1* did cause thymic changes at later time points well after the transition at 28-30 days, such as a premature occurrence of *Atf3*^+^ stress-responsive cTECs and aging-associated TECs in 30-day-old thymi (Fig. 2A-E). Furthermore, the decline in *Foxn1* at the transition in *Foxn1^lacZ^* mutants resulted in a post-transition phenotype in the non-TEC stroma, with unique clusters of endothelium and mesenchyme (Fig. 2F-O), consistent with progressive phenotypes in these mice. We also detected an increase in Wnt signaling in these mutants, beginning at 7 days, which is the time point when *Foxn1* expression is first decreased, but before the transition, suggesting that this change is *Foxn1* dependent but may not be related to the transition itself.

Our intercellular network analysis and unbiased DEG analysis with a logistic regression model strongly suggested that differential expression of *Igf2* and genes in the *Igf2* signaling network were the major source of distinct transcriptomes in endothelium and mesenchyme across the thymic growth transition (Fig. 3A). Thymic capsular and medullary fibroblasts were the main source of *Igf2*, which was mostly received by *Igf1r*-expressing thymic nurse cells, perinatal cTECs and fibroblasts (Figs 3C,D and 4A,B). Interestingly, in the fetal thymus, *Igf2* levels are high early in organogenesis then rapidly decrease after embryonic day 16, then reappear in 0-day-old neonates (Kecha et al., 2000; Kermani et al., 2012). This early IGF signaling has been proposed to promote TEC proliferation during early thymic organogenesis via interactions between thymic mesenchyme-derived IGF1/2 and TEC-derived IGF1R (Jenkinson et al., 2007), suggesting that IGF2 signaling could play a separate role in thymus organogenesis.

This hypothesis is further supported by dynamically regulated IGF receptors and IGF-binding proteins in different subsets of TECs (Fig. 4E-M), even though it is possible IGFBPs can function independently (Allard and Duan, 2018). One of the major IGF interactions we observed was between fibroblasts and cTECs (Fig. 3B-D). Since thymocytes express IGF1R (Fig. S5A,B), IGF2 in the neonatal thymus is also likely to promote thymocyte proliferation, further contributing to neonatal thymic expansion. This possibility is supported by analysis of a murine model with a placental-specific deletion of *Igf2*, resulting in thymic hypoplasia, decreased thymic cellularity and altered thymopoiesis (Bacon et al., 2018). Additionally, *in vitro* experiments show IGF2 is more effective at inducing the proliferation of thymocytes than IGF1 (Kooijman et al., 1995), suggesting an IGF2-specific role in thymocyte proliferation. Our TEC-specific deletion of *Igf1r* further supports an independent role for IGF2 in thymopoiesis through its effect on TECs. T-cell lineage commitment was impaired after TEC-specific *Igf-1R* deletion, suggesting reduced Notch1 signal provided by Delta-like 4 (DLL4), a direct downstream target of *Foxn1* (Žuklys et al., 2016). Interestingly, both accumulation of DN thymocytes and the reduced ratio of MHCII^Hi^/MHCII^Lo^ TECs in Cre^+^ were also observed in the *Foxn1^lacZ^* model itself in a dosage-sensitive fashion (Chen et al., 2009), further supporting IGF signal as an upstream of *Foxn1*.

Our human thymus bulk RNA sequencing data comparison before and after the thymic growth transition also yielded *IGF2* as a top differentially expressed gene (Fig. 7), suggesting an evolutionarily conserved role of IGF2 in driving neonatal thymic expansion. Interestingly, expression of all IGFBP genes decreased after the transition to homeostasis (Fig. 7H-L), while expression of some Igfbp genes increased while others decreased (Fig. 4H-L). As IGFBPs can work both positively and negatively, further functional validations are required to understand their exact roles in the thymus. Interestingly, IGFBP5 is implicated as a marker of aging-associated thymic involution in human thymus, decreasing thymocyte proliferation in aged thymi (Yang et al., 2024). Our data also show a specific increase in *Igfbp5* expression in mouse cTECs at 30 days after birth (Fig. 4J).

**Fig. 8.**
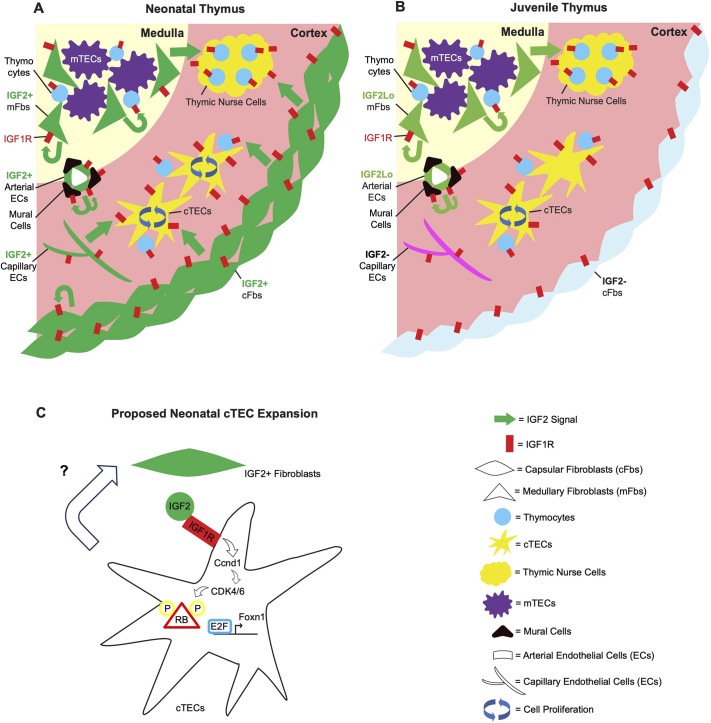
**Hypothetical model: fibroblast-derived IGF2 interacts with IGF1R on cortical thymic epithelial cells, non-thymic epithelial cell stroma and thymocytes to drive neonatal thymic expansion.** Schematics of our hypothetical model. (A) Strong IGF2 signal is coming from non-thymic epithelial cell (TEC) stroma in an autocrine and paracrine fashion, promoting proliferation of cortical TECs (cTECs), non-TEC stroma and thymocytes. (B) After postnatal day 10, the IGF2 signal is diminished and the proliferation of cTECs and non-TEC stroma slows down, resulting in thymic homeostasis. (C) Fibroblast-derived IGF2 interacts with IGF1R. Through cyclin D1-CDK4/6, retinoblastoma (RB) is hyperphosphorylated and detaches from E2F. This enables E2F-mediated transcription of *Foxn1* in cTECs, promoting their proliferation.

Based on our data, we generated schematic diagrams depicting our model for regulation of the transition to homeostasis (Fig. 8). In neonatal thymus, capsular and medullary fibroblasts are the main source of IGF2, sending signals to IGF1R-expressing perinatal cTECs, thymocytes and non-TEC stroma in a paracrine and autocrine fashion. Mitogenic IGF2-IGF1R interactions promote proliferation of perinatal cTECs and thymocytes, resulting in a rapid expansion of the thymic cortex (Fig. 8A). This exponential expansion declines after postnatal day 10 due to significantly decreased *Igf2* transcription in fibroblasts and of *Igf1r* and Igfbp genes in cTECs, with upregulation of inhibitory components of the pathway, resulting in the transition to homeostasis (Fig. 8B).

We propose that this IGF signal acts via E2F-mediated *Foxn1* transcription in cTECs by phosphorylation of RB (Fig. 8C). Our hypothesis is concordant with previous data from a H_2_K^b^ promotor-driven IGF2 overexpression murine model, which exhibits a significantly larger thymic cortex compared to wild-type until 3 months of age, while the thymic medulla remains unchanged in size, resulting in altered thymopoiesis (Kooijman et al., 1995; van Buul-Offers et al., 1995; Van der Ven et al., 1997). This IGF2 overexpression transgenic model poses an interesting question, however, as it does not phenocopy the K5.D1 model (Robles et al., 1996) or the RB-deficient model (Garfin et al., 2013), the thymi of which grow indefinitely. This difference may be due to the decreases in *Igf1r* and Igfbp gene expression, and the rise in IGF2 antagonists, including *Igf2r*, which could prevent transgene-driven IGF2 from affecting cTEC after the transition.

Overall, our study suggests that IGF signaling in both mice and human thymus via IGF2-IGF1R interaction promotes the proliferation of thymocytes, perinatal cTECs, endothelium and fibroblasts, resulting in exponential neonatal thymic expansion prior to the transition to homeostasis at postnatal day 10. Both downregulation of *Igf2* and positive regulators of the pathway, and upregulation of negative regulators occur at the transition, and could drive the transition from growth to homeostasis. Inhibition of IGF2 signaling in TEC prior to the transition affects not only proliferation but also impacts TEC differentiation and thymus function. Comprehensive identification of the IGF family protein and receptor regulation in the thymus could be key to understanding both thymic size expansion and maintenance, as well as function at different ages.

## MATERIALS AND METHODS

### Study design

The objective of this study was to determine molecular mechanisms and/or signaling pathways behind a transition from neonatal expansion to juvenile homeostasis. We used our previously published data for mice and publicly available datasets for humans to determine the transition timing. We used single-cell RNA sequencing of mouse total thymic stroma and bulk RNA sequencing of human thymic tissue to interrogate cellular and gene expression differences before and after the transition. Both murine and human thymic sample information is detailed below. To minimize individual variabilities and technical artifacts, we used three thymi per library with two or three technical replicates for murine scRNAseq. To minimize batch effect, libraries of different conditions were sequenced together whenever possible for murine scRNAseq. To minimize biases, bioinformatics analyses were initially carried out without stratification. For immunohistochemistry, three thymi from different littermates were analyzed to verify the discovery.

### Mice

The *Foxn1^lacZ^* mice were generated and maintained on a C57BL6/J background by the Manley lab (Chen et al., 2009). 129-*Igf1r^tm2Arge^*/J mice (Dietrich et al., 2000) were cryo-recovered from the Jackson Laboratory. All experiments involving animals were performed with approval from the University of Georgia Institutional Animal Care and Use Committee.

### Single-cell suspension of the murine thymic stromal cells for scRNAseq

Our merged scRNAseq dataset contains 3-day-old (three *Foxn1^+/+^* males, three *Foxn1^lacZ/lacZ^* males, two *Foxn1^+/+^* females and two *Foxn1^lacZ/lacZ^* females), 7-day-old (two *Foxn1^+/+^* males and three *Foxn1^lacZ/lacZ^* males) and 14-day-old (two *Foxn1^+/+^* males, two *Foxn1^lacZ/lacZ^* males, two *Foxn1^+/+^* females and two *Foxn1^lacZ/lacZ^* females). Datasets for 30-day-old (two *Foxn1^+/+^* males and three *Foxn1^lacZ/lacZ^* males) are available elsewhere (Xiao et al., 2024 preprint; GSE264402 in Gene Expression Omnibus). Excess blood vessels, connective tissues and adipocytes were carefully removed in ice-chilled 4°C sterile 1×PBS (without Ca^2+^ and Mg^2+^). Thymi were finely cut into ∼1-2 mm^3^ fragments and transferred into digestion buffer [2% FBS/1640 RPMI (with 25 mM HEPES), DNase I (20 µg/µl) and collagenase/dispase (1 mg/ml)]. Tubes containing the thymic fragments with digestion buffer were incubated in a 37°C water bath for 20 min three times. The solution was gently mixed up and down with transfer pipettes in between the incubations, 100 times each. To remove undigested thymic fragments, the solution was passed into a new tube, through a 70 µm cell strainer. The single-cell suspension was then centrifuged for 10 min at 300 ***g***, 4°C, a condition used for centrifugation throughout this protocol. After decanting the supernatant, erythrocytes were lysed with by a 2 min incubation in 2 ml RBC Lysis Buffer (10×) (BioLegend 420301). After another centrifugation and decantation, cells were resuspended in MACS buffer [filtered and degassed 0.5% BSA, 2 mM EDTA and 1×PBS (without Ca^2+^ and Mg^2+^)]. After cell counting with a hemocytometer, cells underwent CD45 separation as per the MACS separation guidelines with mouse CD45 microbeads (Miltenyi Biotec 130-052-301) to collect CD45^−^ cells.

### Single-cell suspension of the murine thymic epithelial cells for scRNAseq

The above methods were used with different digestion buffer [2 ml digestion buffer containing 40 μl of 0.1 mg/ml of Liberase (Sigma-Aldrich) and 20 units/ml of DNAseI (Sigma-Aldrich) in PBS (pH 7.0)]. TECs were collected from postnatal day 0, 3, 7, 10, 15 and 28 wild-type mice. 0.5 ml of digestion buffer was used for perinatal ages ∼0-10 and 2 ml of digestion buffer was used for perinatal ages ∼14-28 per round of digestion. Cells underwent MACS separation to collect CD45^−^ cells. After centrifugation and decantation, CD45 BV510 (BioLegend), EPCAM APC (BioLegend), MHCII APC-Cy7 (BioLegend) and CD11c PerCP-Cy5.5 (Tonbo Biosciences) were added and incubated for 15 min at 4°C. Cells were then washed and resuspended in FACSAria Fusion for sorting. Approximately 30,000-80,000 TECs per time point were submitted for scRNAseq.

### 10X library preparation

Post thymic stromal cell isolation, cell concentration and viabilities were calculated with a hemocytometer and Countess 3 (ThermoFisher Scientific). Libraries were generated using samples with >85% viability and minimal cell clumping. Due to the tendency of thymic stromal cells to adhere to one another, the cells were gently pipetted up and down to avoid further cell clumping. 10,000 cells were loaded into the 10X Genomics Chromium instrument, using a Single Cell 3′ Reagent Kit (v3). After cDNA generation and quantity/quality check steps, the final DNA library was sequenced on Illumina NextSeq2000 P3 with 200 cycles.

### Single-cell transcriptomic data analysis

As per the 10X guidelines, CellRanger (v.6) was used to identify droplets containing cells and create gene-by-cell matrices. Filtered matrices underwent QC steps and downstream analyses were performed using Seurat (v.4.3.0) (Butler et al., 2018; Hao et al., 2021; Satija et al., 2015; Stuart et al., 2019). Low information cells and possible doublets were removed based on thresholds (number of genes>500, UMI<50,000, percent of mitochondrial gene reads<20%). After unsupervised clustering, cell clusters were annotated using known genes. A logistic regression model was used to identify differentially expressed genes for each cluster. Intercellular network analysis was performed with CellChat (v.1.5.0) as per Github guidelines (Jin et al., 2021).

### Human thymus for RNAseq

Human thymus was obtained from anonymous individuals undergoing corrective cardiac surgery where part of the thymus was removed in order to expose the operative field. This tissue was not excised for the purpose of this study and would otherwise have been discarded. The age and sex of the donor were provided, without any other potentially identifying information. The use of anonymous discarded thymus tissue for this research was approved by the Duke University Institutional Review Board (Duke IRB Exempt Protocols 474 and Pro00103028). Samples of each thymus were immediately placed in RNALater (Thermo Fisher Scientific), incubated at 4°C overnight and then stored in liquid nitrogen until used. Frozen tissues were homogenized in a Precellys soft tissue lysing tube (Bertin) and then RNA was extracted using a RNeasy kit (Qiagen). RNA was cleaned and concentrated using Turbo DNA-free (Invitrogen) and RNeasy MiniElute (Qiagen). Libraries were then prepared using a KAPA Stranded mRNA-seq kit (Kapa Biosystems) and sequenced by 100 bp paired-end sequencing on a NovaSeq 6000 (Illumina). For most thymus donors, bulk RNAs derived from at least two separate tissue aliquots were sequenced and the data combined for analysis.

### Bulk transcriptomic data analysis

Quality control on both raw reads and adaptor-trimmed reads was performed using FastQC (www.bioinformatics.babraham.ac.uk/projects/fastqc). RNA reads were aligned to the reference genome hg38 using STAR (Dobin et al., 2013) and quantified by featureCounts (Liao et al., 2013). DESeq2 (Anders and Huber, 2010; Love et al., 2014) was used to detect differential expression between younger (<3-month-old) and older (≥3-month-old) samples, considering sex as a confounding factor. Benjamini-Hochberg correction was applied to adjust for multiple testing. Functional enrichment analysis was performed by GSEA (Subramanian et al., 2005). Principal component analysis was used to show variations across samples.

### Immunohistochemistry

After the thymus was harvested from an animal, connective tissues and excess blood vessels were removed from the thymus in ice-cold sterile Ca^2+^, Mg^2+^-free 1×PBS. The thymus was then fixed in 4% PFA/1×PBS for 2 h on ice. After washing away 4% PFA/1×PBS, the thymus was incubated in 20% sucrose (with 0.05% NaN_3_) at 4°C, overnight. The following day, the thymus was then placed in an O.C.T. mold filled with O.C.T. to be snap-frozen with dry ice. Frozen sections (10 µm) of murine thymus on slides were prepared with a cryostat. ∼100 µm of murine thymus was discarded to capture the whole thymus with proper cortical-medullary structure. The slides were then acclimatized at room temperature for 30 min. After removing residual O.C.T. on the slide with sterile Ca^2+^- and Mg^2+^-free 1×PBS, the sections were blocked with 10% donkey serum diluted in 0.5% BSA and 0.2% Tween-20 in 1×PBS for 30 min. After blocking, sections were incubated in a solution containing optimally diluted primary antibodies at 4°C, overnight. The following day, the sections were washed in sterile Ca^2+^- and Mg^2+^-free 1×PBS. The sections then were incubated in fluorochrome-conjugated secondary antibodies for 1 h at room temperature. After washing, the coverslips were applied and a Zeiss LSM880 was used to acquire microscope images.

Formalin-fixed, paraffin-embedded (FFPE) sections of thymus tissue from human infants 4-23 days (*n*=7) and 4.7-6 months (*n*=5) of age were reacted with IGF2 mAb 8H1 (Invitrogen, MA5-17096), fibroblast mAb TE7 (Chemicon) or mouse IgG (Dako, X0931) using standard immunoperoxidase techniques. Slides were deparaffinized, blocked with 2% hydrogen peroxide in methanol for 10 min, heated in 10 mM citrate buffer (pH 6.0) using a pressure cooker, cooled for 20 min, then sequentially incubated with goat serum for 30 min and a 1:200 dilution of 8H1 for 1 h, all at room temperature. After three washes, the HRP-labeled polymer detection system (Dako, K4001) was applied for 45 min, washed three times and the color developed using a 5 min incubation with 3,3′-diaminobenzidine substrates, followed by counterstaining with hematoxylin. Brown color indicates positive reaction with antibody.

### Immunohistochemistry – antibodies

The following antibodies were used: anti-APOD (Invitrogen, PA5-27386; 1:200), anti-VEGFR2 (Invitrogen, MA5-15157; 1:200), murine anti-IGF2 (Invitrogen, PA5-47946; 1:40), human anti-IGF2 (Invitrogen, MA5-17096; 1:200), anti-IGF1Rß (Invitrogen, PA5-37601; 1:100), anti-CD3 (Invitrogen, MA1-80783; 1:200), anti-CD205 (BioLegend, 138201; 1:200), donkey anti-goat IgG (H+L) Cross-Adsorbed Secondary Antibody, Alexa Fluor 488 (Invitrogen, A-11055; 1:400), donkey anti-rat IgG (H+L) Cross-Adsorbed Secondary Antibody, Alexa Fluor 594 (Invitrogen, A-21209; 1:400) and donkey anti-rabbit IgG (H+L) Cross-Adsorbed Secondary Antibody, Alexa Fluor 647 (Invitrogen, A-31573; 1:400).

### Flow cytometry

Freshly isolated thymocytes from 0.5- to 1 day-old pups (∼1×10^6^) were used for each sample. Cells were blocked using anti-CD16/32 (Clone:93) antibody before staining. For phenotypic profile and counting the numbers of thymocyte subsets, anti-CD4 APC-Cy7, anti-CD8 PerCp, anti-CD44 FITC (IM7) and anti-CD25 APC (3C7) were used. For analysis of thymic progenitor Lin^−^ DN1a,b T cells in the total DN1 subpopulations, phycoerythrin (PE)-conjugated lineage markers, including anti-CD3 (145-2C11) (BioLegend, 100307; 1:200), anti-CD4 (BioLegend, 100307; 1:200), anti-CD8 (BioLegend, 100307; 1:200), anti-CD11c (N418) (BioLegend, 117307; 1:200), anti-CD19 (BioLegend, 115508; 1:200), anti-Gr-1 (RB68-C5) (BioLegend, 108407; 1:200), anti-TER-119 (TER-119) (BioLegend, 116207; 1:200) and anti-NK1.1 (BioLegend, 108708; 1:200) antibodies were mixed and combined with anti-CD25 APC (BioLegend, 102012; 1:200) and anti-CD44 PE-Cy7 (BioLegend, 103029; 1:200), anti-CD117 APC-Cy7 (2B8) (BioLegend, 105825; 1:200) and anti-CD24 FITC (M1/69) (BioLegend, 101805; 1:200) antibodies.

For TEC isolation, thymic lobes from 0.5-1 days old pups were cut once or twice and gently washed in 2% FBS RPMI 1640 medium to remove thymocytes. The thymic pieces were digested in 5 ml of collagenase/dispase (1 mg/ml, Roche) plus DNase I (20 ng/ml, Sigma) in 2% FBS RPMI 1640 medium, placed in a 37°C water bath for 60 min, and agitated by passing through an 18 G needle five times and a 25 G needle twice. Cells were then filtered by passing through a 70 μm cell strainer. For TEC phenotypical analysis, the digested cells (1-2×10^6^) were incubated with anti-CD45-PE-Cy7 (30-F11) (BioLegend, 103113; 1:200), anti-MHCII-PerCp (M5/114.15.2) (BioLegend, 107623; 1:200) or anti-EpCAM-APC (G8.8) (BioLegend, 118213; 1:200) on ice for 20 min. Cells were washed and then placed in a 1% PFA PBS solution for analysis.

### Flow cytometry – statistical analysis

Data points were inserted and analyzed using Prism software (GraphPad Software) with an unpaired Student's *t*-test.

## Supplementary Material



10.1242/develop.204347_sup1 Supplementary information
