# Supplementary material for: Insulin-like growth factor 2 as a driving force for exponential expansion and differentiation of the neonatal thymus
Source: Development. 2025 Apr 10;152(7):dev204347. doi: 10.1242/dev.204347 (PMC12045631; doi:10.1242/dev.204347)
Supplement: Supplementary information [file develop-152-204347-s1.pdf]

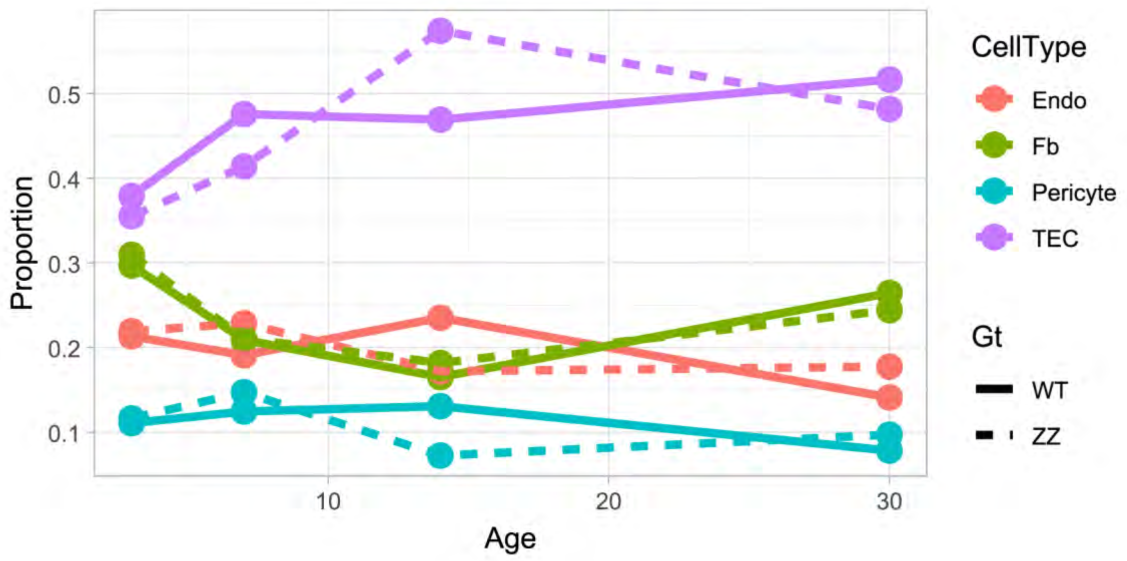

Fig. S1. Total murine thymic stroma proportional change across time. Line graph shows proportional change of total murine thymic stroma across time, calculated from our time-series scRNAseq dataset. Each subset is color-coded & separated by genotype: *Foxn1*$^{+/+}$ & *Foxn1*$^{Z/Z}$. Age: postnatal days.

Development • Supplementary information

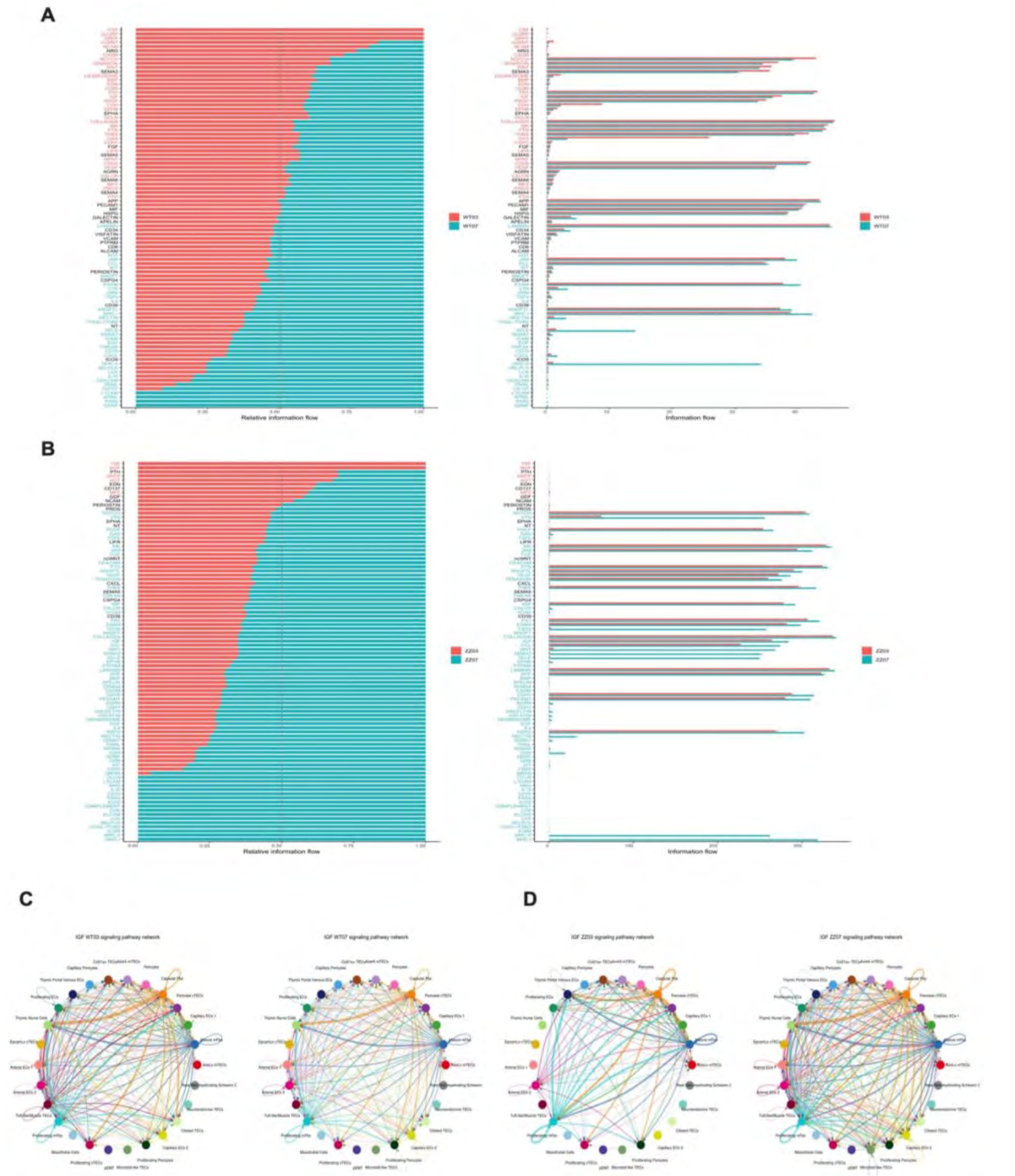

Fig. S2. Intercellular network analysis between genotype-matched 3- and 7-day-old samples.
(A) Bar graphs showing differentially expressed intercellular interaction pathway comparisons between 3-day-old and 7-day-old *Foxn1*$^{+/+}$ samples. (B) Bar graphs showing differentially expressed intercellular interaction pathway comparisons between 3-day-old and 7-day-old *Foxn1*$^{lacZ/lacZ}$ samples. (C, D) Circle plots showing comparisons between genotype-matched 3-day-old and 7-day-old samples.

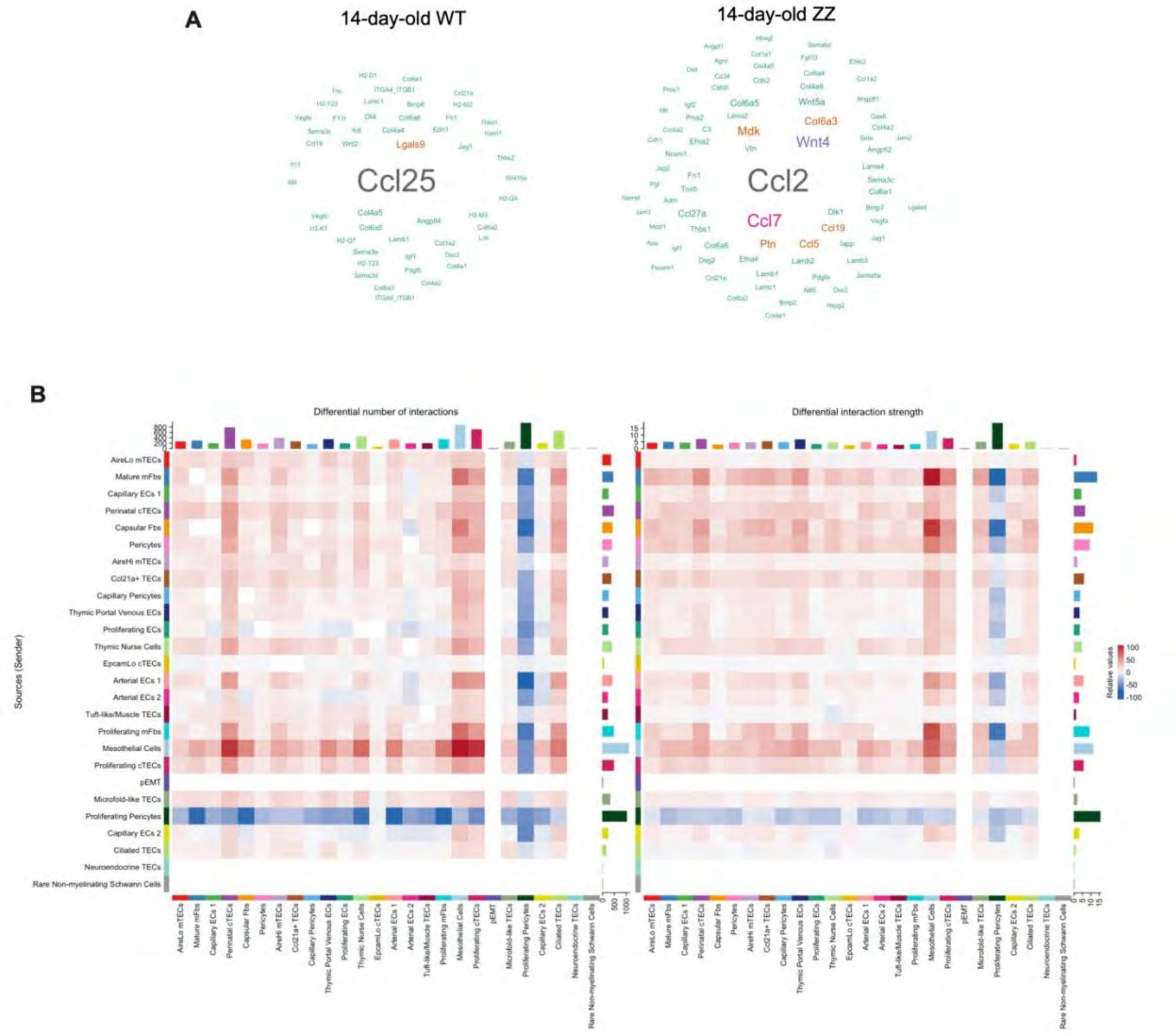

Fig. S3. Intercellular interaction comparison between 14-day-old *Foxn1*$^{+/+}$ and *Foxn1*$^{lacZ/lacZ}$.

(A) Ligand enrichment comparison of 14-day-old *Foxn1*$^{+/+}$ and *Foxn1*$^{lacZ/lacZ}$. (B) A heat map showing all the interactions amongst different cell types. The Y-axis indicates the clusters expressing ligands; the X-axis indicates the clusters expressing receptors. Computed interaction strength scales show substantially reduced overall intercellular interactions in 14-day-old *Foxn1*$^{lacZ/lacZ}$.

Development • Supplementary information

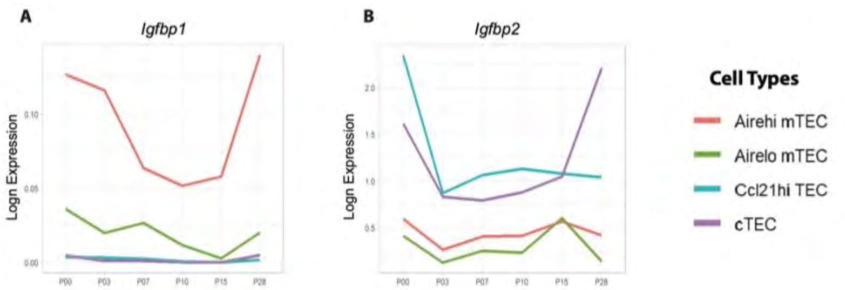

Fig. S4. *Igfbp1* & *Igfbp2* expression in perinatal TECs.

(A, B) Line graphs show average expression of *Igfbp1* and *Igfbp2* across time in different TEC subsets. Figure legends are located at the right.

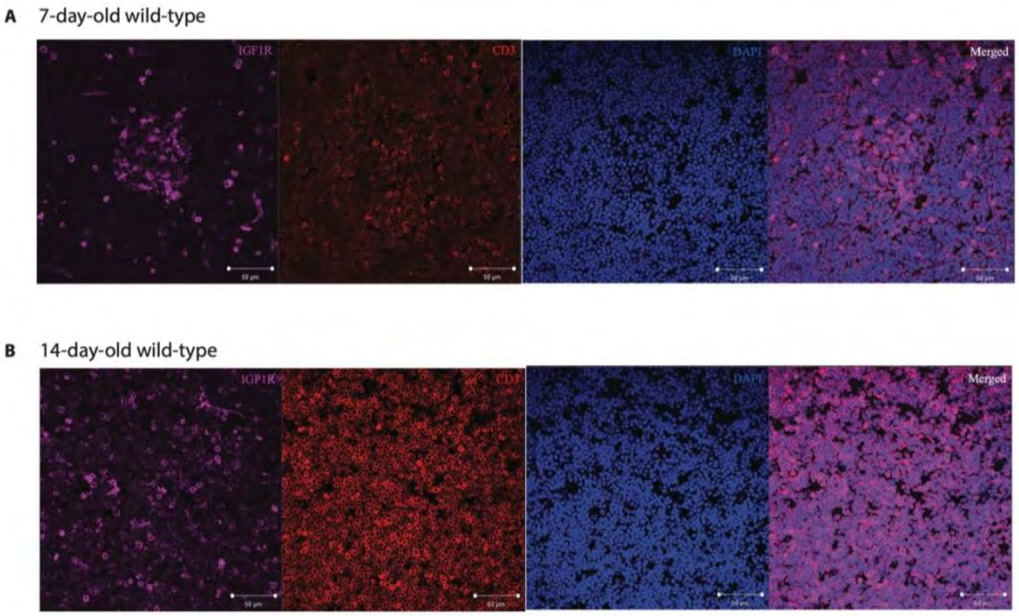

Fig. S5. Thymocytes expressing IGF1R.

(A) Immunohistochemistry of frozen 7-day-old *Foxn1*$^{+/+}$ thymic sections (10 µm). The first panel is IGF1R (magenta), the second is CD3 (red), the third is DAPI (blue), and the fourth is merged. (B) Immunohistochemistry of frozen 14-day-old *Foxn1*$^{+/+}$ thymic sections (10 µm). The first panel is IGF1R (magenta), the second is CD3 (red), the third is DAPI (blue), and the fourth is merged. Many CD3+ cells are IGF1R+. Of note, the CD3 protein level is visibly increased after postnatal day 10. Scale bars = 50µm.

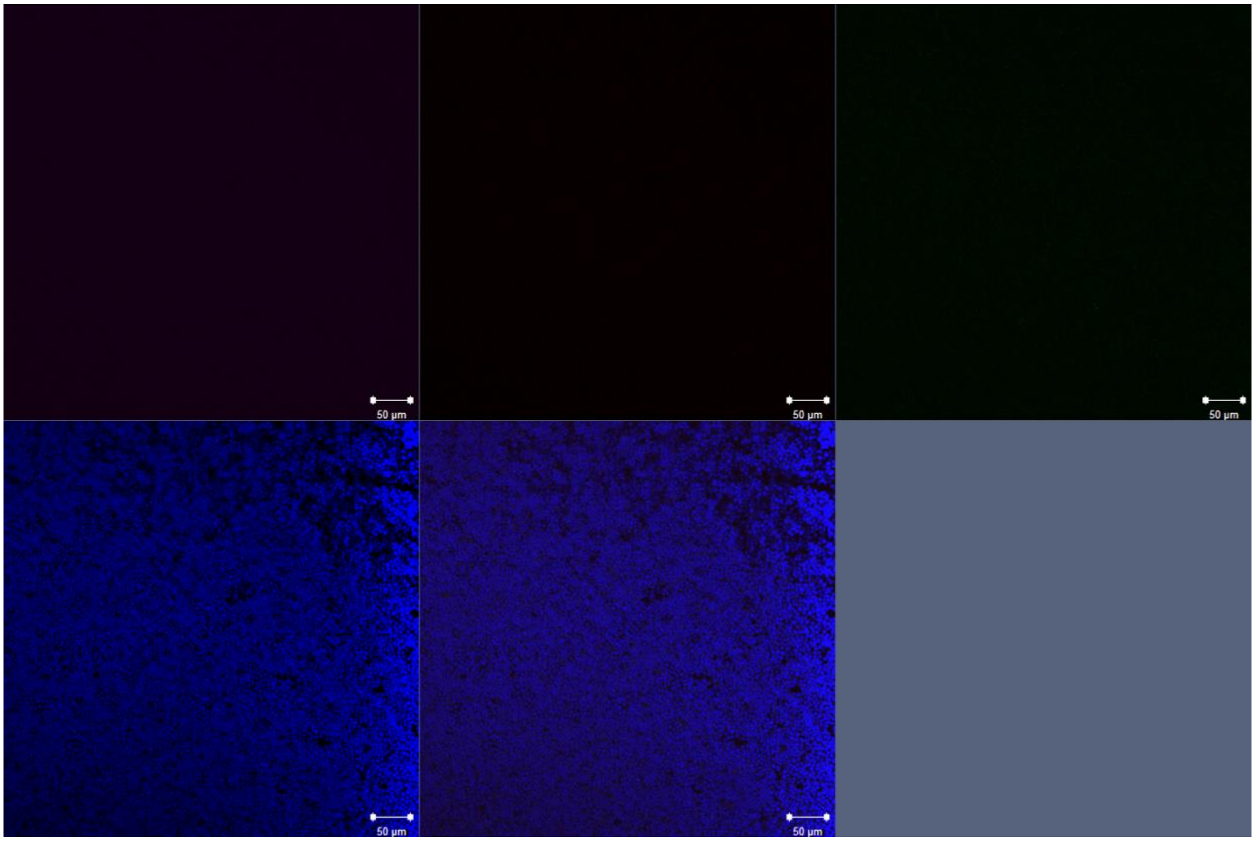

Fig. S6. Negative controls of antibody staining.

Negative controls of α-IGF2 (Invitrogen, Cat. # PA5-47946), α-CD205 (BioLegend, Cat. # 138201), α-APOD (Invitrogen, Cat. # PA5-27386) with DAPI staining. 7-day-old male *Foxn1*$^{+/+}$ sample. Scale bars = 50µm.

Development • Supplementary information

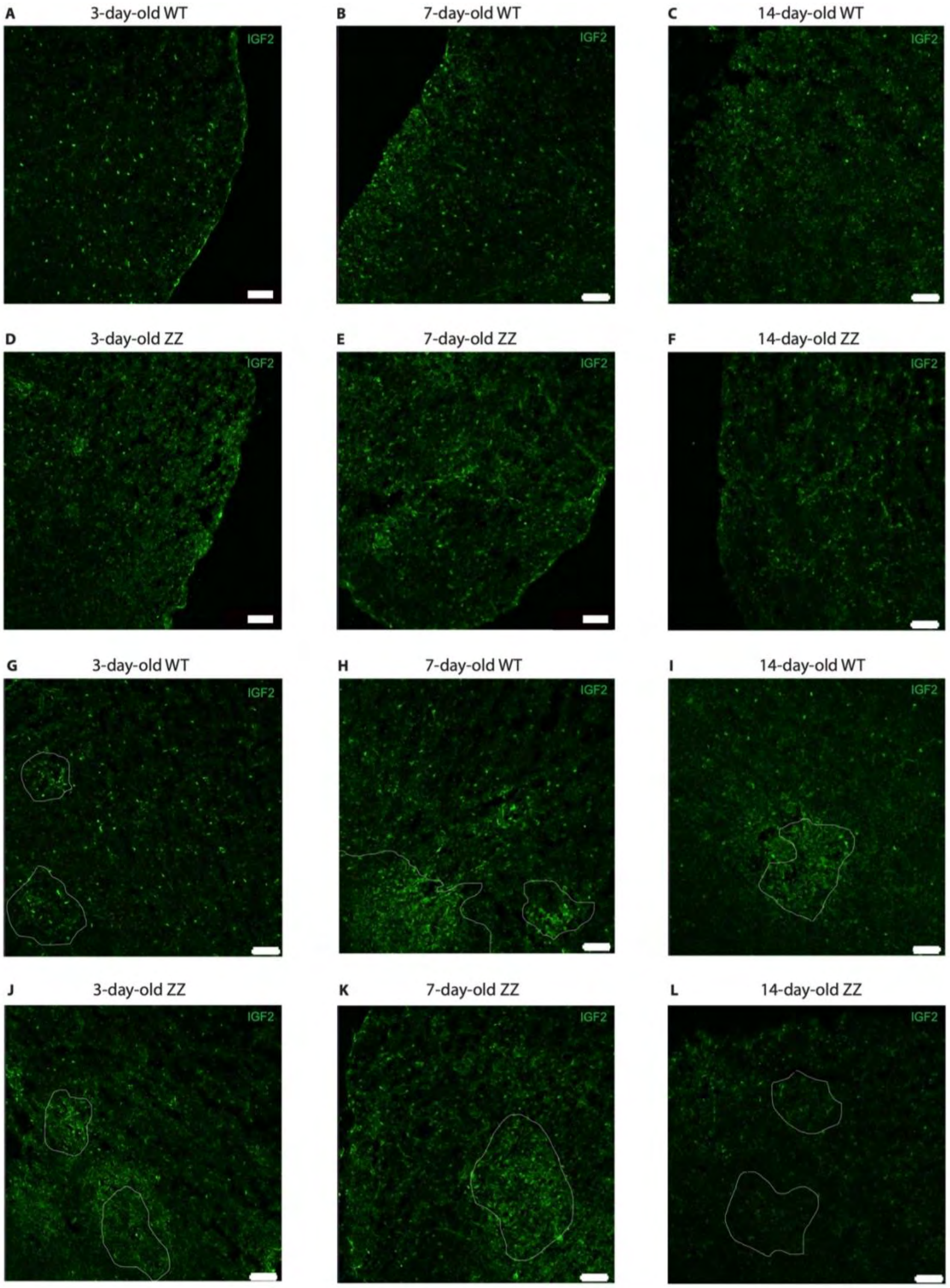

Fig. S7. Cortical and medullary expression of IGF2. Immunohistochemistry (IHC) of frozen thymic sections (10 µm). (A-C) IGF2 distribution in 3-, 7-, and 14-day-old *Foxn1*$^{+/+}$ males. (D-

Development • Supplementary information

F) IGF2 distribution in 3-, 7-, and 14-day-old *Foxn1*$^{lacZ/lacZ}$ males. (G-I) IGF2 distribution in 3-, 7-, and 14-day-old *Foxn1*$^{+/+}$ males. Medulla was circled based on CD205 and DAPI expression. (J-L) IGF2 distribution in 3-, 7-, and 14-day-old *Foxn1*$^{lacZ/lacZ}$ males. Medulla was circled based on CD205 and DAPI expression (S. Fig. 6). WT: *Foxn1*$^{+/+}$, ZZ: *Foxn1*$^{lacZ/lacZ}$. Scale bars = 50 µm.

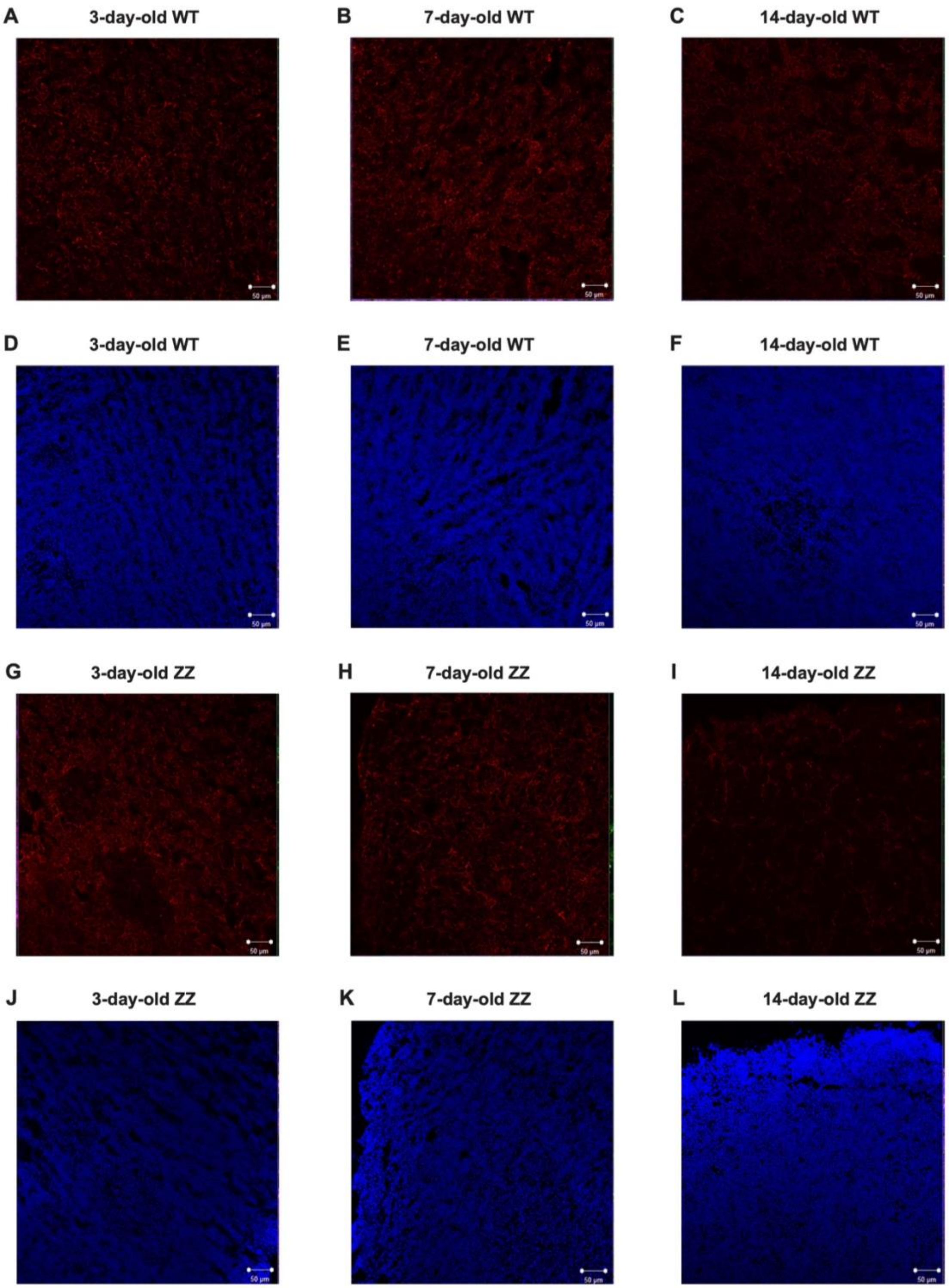

Fig. S8. Thymic cortex distinction with CD205 and DAPI. Immunohistochemistry (IHC) of frozen thymic sections (10 µm), correspond to S. Fig. 5G-L. (A-C, G-I) CD205 expression in 3-, 7-, and 14-day-old *Foxn1*$^{+/+}$ and *Foxn1*$^{lacZ/lacZ}$ males. (D-F, J-L)
DAPI expression in 3-, 7-, and 14-day-old *Foxn1*$^{+/+}$ and *Foxn1*$^{lacZ/lacZ}$ males. WT: *Foxn1*$^{+/+}$, ZZ: *Foxn1*$^{lacZ/lacZ}$. Scale bars = 50 µm.

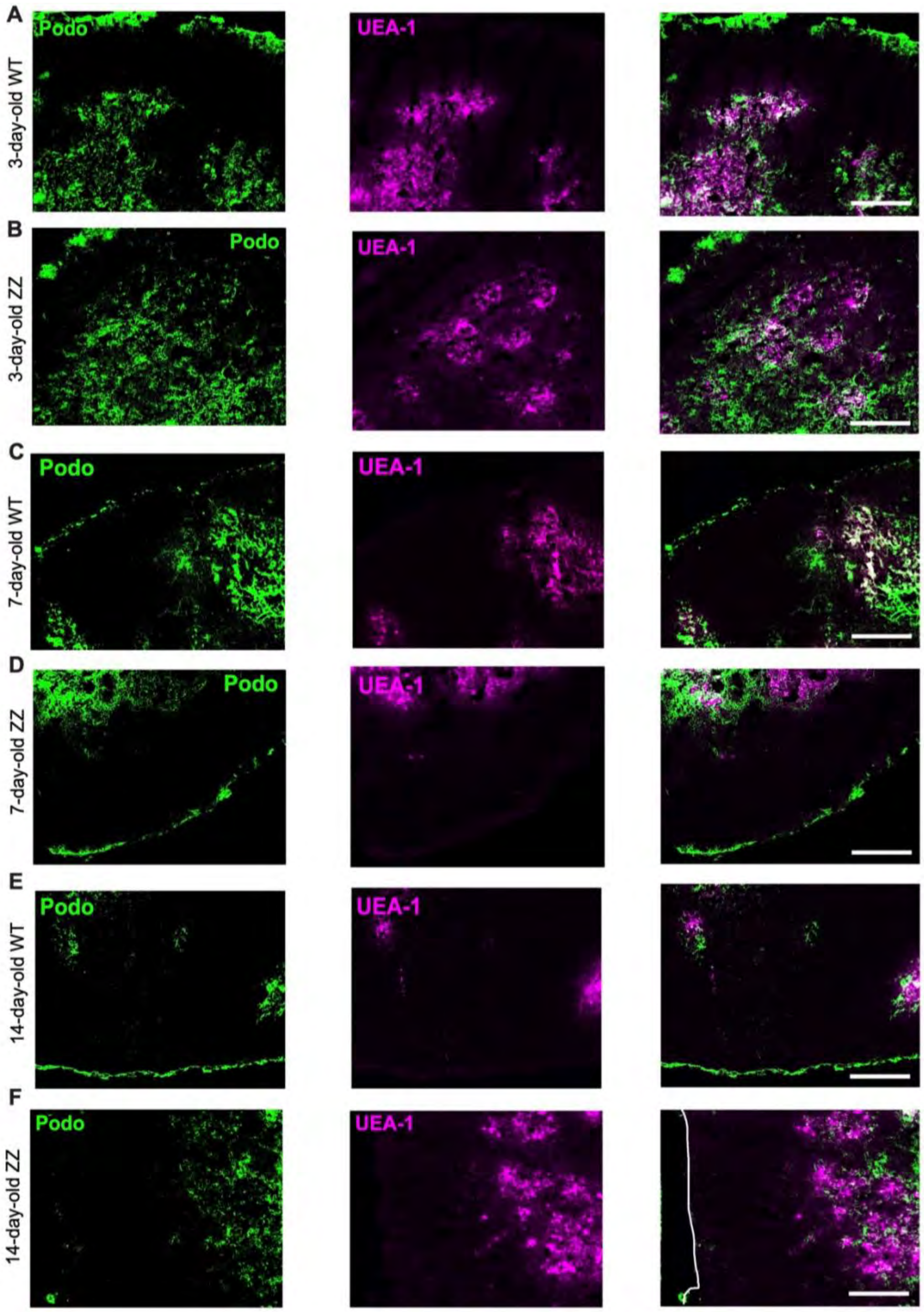

Fig. S9. Thymic capsular fibroblasts (cFbs) across age of *Foxn1*$^{+/+}$ and *Foxn1*$^{lacZ/lacZ}$. Immunohistochemistry (IHC) of frozen thymic sections (10 µm). (A-F) Podoplanin (Podo) and UEA-1 (mTECs) expressions in 3-, 7-, and 14-day-old *Foxn1*$^{+/+}$ and *Foxn1*$^{lacZ/lacZ}$ males. White line on S. Fig. 8F shows the thymic capsule. WT: *Foxn1*$^{+/+}$, ZZ: *Foxn1*$^{lacZ/lacZ}$. Scale bars = 100 µm.

Development • Supplementary information

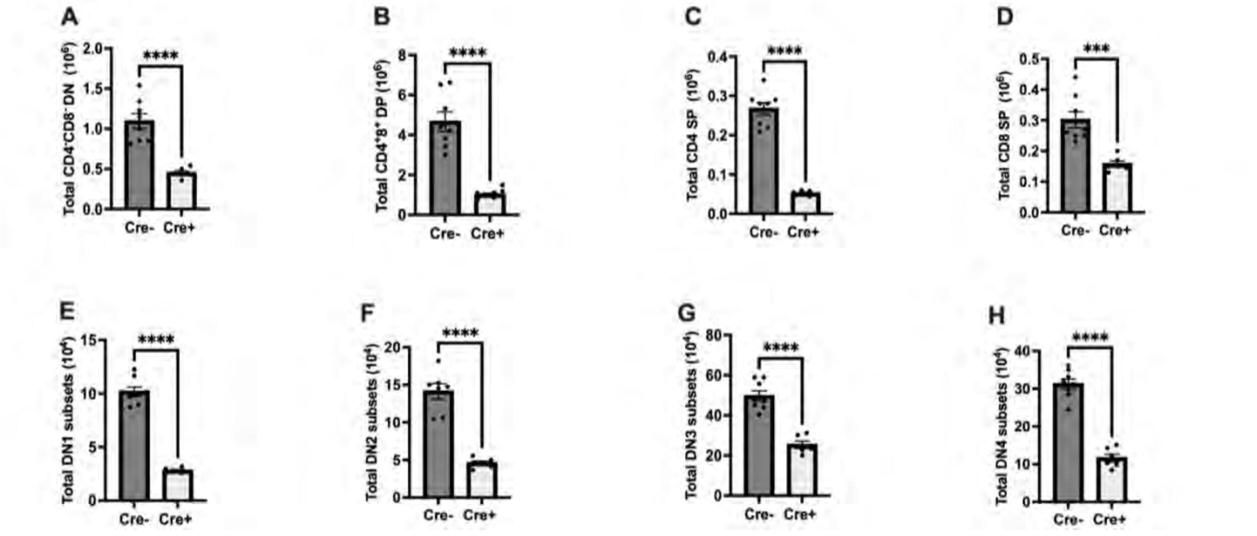

Fig. S10. TEC-specific IGF1R deletion results in reduced thymocyte number.

(A-D) Reduced number in CD4-CD8- DN, CD4+CD8+ DP, CD4+ SP, CD8+ SP thymocytes in TEC-specific IGF1R knock-out mice. (E-H) Reduced number in DN1-4 thymocytes in TEC-specific IGF1R knock-out mice. DN: double-negative, DP: double-positive, SP: single-positive, Cre-: *Foxn1*$^{+/+}$ X Igf1r$^{tm2Arge/tm2Arge}$, Cre+: *Foxn1*$^{Cre/+}$ X Igf1r$^{tm2Arge/tm2Arge}$.

Development • Supplementary information
