# Supplementary material for: Insulin-like growth factor 2 as a driving force for exponential expansion and differentiation of the neonatal thymus
Source: Development. 2025 Apr 10;152(7):dev204347. doi: 10.1242/dev.204347 (PMC12045631; doi:10.1242/dev.204347)
Supplement: Supplementary file 1 [file develop-152-204347_review_history.pdf]

Insulin-like growth factor 2 as a driving force for exponential expansion and differentiation of the neonatal thymus

Seung Woo Kang, Bryan R. Helm, Yu Wang, Shiyun Xiao, Wen Zhang, Anusha Vasudev, Ken S. Lau, Qi Liu, Ellen R. Richie, Laura P. Hale and Nancy R. Manley

DOI: 10.1242/dev.204347

Editor: Anna-Katerina Hadjantonakis

Review timeline

Original submission: 22 August 2024
Editorial decision: 12 October 2024
First revision received: 18 February 2025
Accepted: 9 March 2025

Original submission

First decision letter

MS ID#: dev.204347

MS TITLE: Insulin-like growth factor 2 (IGF2) as a driving force of exponential expansion in neonatal thymus

AUTHORS: Nancy Manley; Seung Woo Kang; Bryan R Helm; Yu Wang; Ken S Lau; Qi Liu; Ellen R Richie; Laura P Hale

I have now received all the referees reports on the above manuscript, and have reached a decision. The referees' comments are appended below, or you can access them online: please go to.

The overall evaluation is positive and we would like to publish a revised manuscript in Development, provided that the referees' comments can be satisfactorily addressed. Please attend to all of the reviewers' comments in your revised manuscript and detail them in your point-by-point response. If you do not agree with any of their criticisms or suggestions explain clearly why this is so. If it would be helpful, you are welcome to contact us to discuss your revision in greater detail. Please send us a point-by-point response indicating your plans for addressing the referees' comments, and we will look over this and provide further guidance.
attend to all of the reviewers' comments in your revised manuscript and detail them in your point-by-point response. If you do not agree with any of their criticisms or suggestions explain clearly why this is so. If it would be helpful, you are welcome to contact us to discuss your revision in greater detail. Please send us a point-by-point response indicating your plans for addressing the referees' comments, and we will look over this and provide further guidance.

Reviewer 1

SUMMARY OF THE ADVANCE MADE IN THIS PAPER AND ITS POTENTIAL SIGNIFICANCE TO THE FIELD

The manuscript by Kang et al. addresses the mechanisms regulating neonatal thymus growth and development using a transcriptomic approach, with a particular focus on the role of Foxn1 in these processes. Given the distinct role of the neonatal thymus in establishing lifelong T cell immunity,

© 2025. Published by The Company of Biologists under the terms of the Creative Commons Attribution License (https://creativecommons.org/licenses/by/4.0/).

this is an important area of inquiry. The authors identify IGF2 is a potential critical paracrine signal regulating thymus growth in the neonatal period.

The study includes robust approaches, and the data presented strongly support the claims made by the authors. A few minor concerns and suggestions are included below, but they are not intended to be viewed as necessary revisions.

SUGGESTIONS TO AUTHORS

For figure 3, more information regarding the CellChat approach would be helpful in interpreting the results. For instance, does "IGF signal" distinguish between IGF1 and IGF2 or include both, or only common factors? A brief description of how CellChat works and how it was used here (e.g, version number) in the methods section would be helpful to investigators interested in replicating the study.

In reference to the results in figure 4H-L and 4I-J the authors refer to the data as suggesting changes in the "aged" thymus (eg line 284), but since the oldest mice are 28 days of age, it may be more appropriate to refer to changes as characterizing the growth to homeostasis transition as they do elsewhere.

The human IGF2 staining in figure 6 N-O seems relatively uniform, perhaps non-specific, and it would increase confidence to display a negative control, and/or to show a fibroblast marker stain in these panels.

Reviewer 2

In this study, Kang et al. investigate the molecular changes occurring in distinct components of the thymic stroma, including thymic epithelial cells, endothelial cells, and mesenchymal cells, with the aim of identifying novel pathways involved in the growth of the thymus during early postnatal life. The team begin by providing a compelling characterization of single-cell transcriptomic alterations in these stromal components in both wild-type (WT) mice and a previously described model with altered Foxn1 expression (Foxn1lacZ/lacZ), which in itself offers an interesting framework for researchers in the field (Figures 1-2). The initial objective appeared to be exploring the role of Foxn1 and its targets in driving the thymic growth during early postnatal development.
The authors describe several alterations across the different epithelial, mesenchymal, and endothelial components, which become more pronounced and Foxn1-dependent as the thymus transitions into adulthood. Despite these later-life changes, the authors shift their attention to the first few weeks of postnatal life, noting that thymic growth during this early period (days 3, 7, and 14) describing to be Foxn1-independent, based on the seemingly identical profile of WT and Foxn1 mutant mice.
Exploring bioinformatic approaches to identify potential pathways involved in the early growth phase, the authors highlight IGF2 signaling as a possible candidate (Figure 3). Further analysis attempted to reveal the likely source of IGF2 as mesenchymal cells and its receptors located on thymic epithelial cells (TECs) (Figures 4-5), suggesting a potential IGF2-mediated mesenchymal-epithelial interaction. The manuscript concludes by demonstrating the expression of IGF2 in the human thymus (Figure 6).

While the scope of the manuscript is intriguing and the observations are of value, there are several aspects that should be addressed to strengthen the conclusions. These include further validation of the bioinformatic predictions, improved analysis of IGF2 in mesenchymal cells as well as additional functional assays to confirm the role of IGF2 signaling in TECs. Addressing these points would enhance the overall impact and robustness of the study's findings.

Specific points:

1. As a starting point, the authors should consider to include and/or discuss how the cellularity of TECs, endothelial, and mesenchymal cells evolve during the analyzed temporal window (d3-7-14-

© 2025. Published by The Company of Biologists under the terms of the Creative Commons Attribution License (https://creativecommons.org/licenses/by/4.0/).

30). This would provide valuable insights into the expansion rates of these populations, offering a clearer understanding of the early thymic growth for the general reader.

2. The panels A-C in Figure 1 are complex to interpret at first glance. Given the clearer presentation and deconvolution of thymic stroma populations in panels D-G and Figure 2, it may be better to either move panels A-C to the supplementary section or omit them entirely. This would streamline the main figures and enhance the overall clarity of the manuscript.

3. The evidence that Foxn1-dependent changes in TECs after day 14 lead to alterations in the diversity of TECs, mesenchymal, and endothelial cells (Figure 2 e.g. aaTEC, capillary ECs, mature medullary fibroblasts, in WT vs mut) is only superficially described and not thoroughly explored. While the review acknowledges that this may be the focus of another study, the authors should consider highlighting and discussing these alterations more explicitly.

MAIN: While the analysis suggests a potential involvement of IGF2, the conclusions, including the title, are overstated and require some attention. The data may suggest IGF2 as a possible factor, but further validation is needed to make definitive claims.

4a. Ligand enrichment analysis (Fig3 A). Do the authors also conduct similar analysis on day 3? Thymic expansion is a dynamic process. Is this purported signaling pathway already present on day 3?

4b The analysis of IGF2 in immunofluorescence and the possible expression in mesenchymal cells (capsular fibroblast) is not very easy to appreciate and entirely convincing, mostly related with the co-localization of IGF2 staining and capsular fibroblasts (Fig5). The authors may attempt to better identify capsular and medullary fibroblast using other markers (e.g. gp38+ DPP4+) (Nitta et al., 2020),

4c. Can the authors provide functional evidence for a possible role of IGF2 in TEC? For example FTOCs/ or culturing ex vivo isolated TECs culture with recombinant IGF2. Assessment of proliferation, Foxn1 levels may be measured in these conditions.

4d. The authors conclude from the results in Figure 6 that IGF2 expression increases in the human thymus. However, the results actually show a decrease in expression between the periods before and after 3 months, as well as between the thymus at 7 days old and at 13.5 months. Rather than an increase, we observe a decrease in expression between the younger and older thymus samples. The absence of an earlier time point, with lower expression of IGF2, prevents to claim that IGF2 increases in the neonatal thymus.

4e. Previous scRNA-seq studies of the human thymus have characterized distinct thymic stromal components (Park et al., Science 2020; Bautista et al., Nat Comm 2021). Could the authors analyze these available datasets to map IGF2 expression in human mesenchymal subsets and generate a similar representation to that shown in Figure 4 for the mouse datasets?

Reviewer 3

SUMMARY OF THE ADVANCE MADE IN THIS PAPER AND ITS POTENTIAL SIGNIFICANCE TO THE FIELD

Overall, this is an interesting paper which sets up a mechanistic model to explain regulation of the transition from fetal growth to juvenile homeostasis during thymus development, that occurs at approximately P10 in mice. The authors first examine and appear to negate a smoking gun hypothesis, that this transition may be regulated by modulated Foxn1 expression levels. They then analyse transcriptional changes in thymic stromal cell types to identify IGF2 expression in thymic capsular fibroblasts as a strong candidate. Further analysis indicates tight regulation of IGF signalling in the developing thymus, with upregulation of the inhibitory receptor Igfr2 plus several Igfbps coinciding with the downregulation of Igf2 and the transition to homeostasis. Analysis of human datasets suggest conservation of this mechanism, corroborated by immune-staining. Finally, the authors present a model of how IGF2 expression in subcapsular and medullary fibroblasts, and

© 2025. Published by The Company of Biologists under the terms of the Creative Commons Attribution License (https://creativecommons.org/licenses/by/4.0/).

arteriolar endothelial cells, likely regulates thymic epithelial cell (TEC)(and hence thymus) growth, including through stimulation of Cyclin D1 expression leading to RB hyperphosphorylation and thus both cell cycle progression and elevated Foxn1 expression. The manuscript is well written and the data well presented. The novel scRNAseq datasets included represent a substantial resource for the field, that will yield information beyond the focus of the current study. Functional testing of the IGF2 model is not presented, but some previous studies are relevant here and could be discussed - ameliorating this concern to some extent. Additionally, exploration of the mechanism leading to Igf2 (and Igfr1) downregulation would be welcome, as would be analysis of the effect of Foxn1 downregulation in non-TEC stroma the Foxn1Z mice (although this occurs after the transition under study it seems an omission to omit it here).

SUGGESTIONS TO AUTHORS
Issues that must be addressed
There have been a number of previous studies describing effects of IGF2 overexpression on the thymus in addition to the Van der Ven paper already discussed (including the initial characterisation of the H2-Kb-IGF-II transgenic line). These papers provide further evidence relevant to the model presented and could usefully be considered in the discussion (e.g. Koojman et al PMID: 7751625 show effects on thymus cellularity consistent with the predictions of the current hypothesis, and van Buul-Offers et al PMID: 7537791 provide some preliminary findings on the effects of elevated IGF2 levels on thymus size). There is also some relevant early work from the Savino and Geenan (e.g. https://doi.org/10.1210/endo.141.3.7360) labs, that again could usefully be considered in the discussion. The authors are requested to consider and discuss these papers (even briefly) in the context of the model they propose.

Related, the model proposed is complex, with effects of IGF2 likely on TEC and thymocytes. Are further clues (beyond changes in IGF pathway components) as to which compartment is primarily affected to cause the growth to homeostasis transition evident in the scRNAseq data (or are both equally affected), and as to the cause of IGF2 downregulation? Exploration and/or more extended discussion of this should be included.
Additionally, the data related to postnatal downregulation of Foxn1 in the Z/Z mice should be considered and presented in more depth. The authors currently deal with this in quite a cursory manner, noting that overall TEC phenotypes do not appear to have changed in the Z/Z mice at P14, while changes at P30 are consistent with a more recent report. However, in the original publication, Z/Z mice were shown to have smaller thymi by P14, presumably resulting from changes in the TEC compartment. Since the Z/Z mice appear from the original publication effectively not to go into homeostasis at this transition, further analysis of the Z/Z datasets to show the major impacts of reduced Foxn1 in the TEC compartment by P14 and on the non-TEC stroma at P14 and P30 should be presented. I appreciate that the authors may consider this separate to the focus of the paper, but since the scRNAseq are included in this study and the timeframe is related, it should be included here at least as supplementary information.

Minor issues
Line 71 - define Foxn1 in the previous sentence.
Line 88 - 'Instead' not correct here - instead of what?
Line 205 - good to add in a sentence reiterating that clear Foxn1-dependent changes were also observed but occurred after the P10 transition.

Fig 5 - Please show negative controls for IGF2 staining (as a supplementary figure), and comment on the source of the staining seen throughout the cortex. Is IGF2 expressed by thymocytes?

SF3 - Please show corresponding CD205 and DAPI stains.

First revision

Author response to reviewers' comments

© 2025. Published by The Company of Biologists under the terms of the Creative Commons Attribution License (https://creativecommons.org/licenses/by/4.0/).

Reviewer 1: SUMMARY OF THE ADVANCE MADE IN THIS PAPER AND ITS POTENTIAL SIGNIFICANCE TO THE FIELD

The manuscript by Kang et al. addresses the mechanisms regulating neonatal thymus growth and development using a transcriptomic approach, with a particular focus on the role of Foxn1 in these processes. Given the distinct role of the neonatal thymus in establishing lifelong T cell immunity, this is an important area of inquiry. The authors identify IGF2 is a potential critical paracrine signal regulating thymus growth in the neonatal period.

The study includes robust approaches, and the data presented strongly support the claims made by the authors. A few minor concerns and suggestions are included below, but they are not intended to be viewed as necessary revisions.

SUGGESTIONS TO AUTHORS

* For figure 3, more information regarding the CellChat approach would be helpful in interpreting the results. For instance, does "IGF signal" distinguish between IGF1 and IGF2 or include both, or only common factors? A brief description of how CellChat works and how it was used here (e.g, version number) in the methods section would be helpful to investigators interested in replicating the study.

IGF signal does distinguish between IGF2-IGF1R & IGF1-IGF1R interactions.
However, *Igf1* expression is barely detectable until postnatal day 28.
To address the concern, we specified IGF2-IGF1R interactions in the results & figure legends.

* In reference to the results in figure 4H-L and 4I-J the authors refer to the data as suggesting changes in the "aged" thymus (eg line 284), but since the oldest mice are 28 days of age, it may be more appropriate to refer to changes as characterizing the growth to homeostasis transition as they do elsewhere.

Suggestions reflected in the results section.

* The human IGF2 staining in figure 6 N-O seems relatively uniform, perhaps non-specific, and it would increase confidence to display a negative control, and/or to show a fibroblast marker stain in these panels.

We added negative control panels & fibroblast figure panels to Figure 7.

Reviewer 2: In this study, Kang et al. investigate the molecular changes occurring in distinct components of the thymic stroma, including thymic epithelial cells, endothelial cells, and mesenchymal cells, with the aim of identifying novel pathways involved in the growth of the thymus during early postnatal life.
The team begin by providing a compelling characterization of single-cell transcriptomic alterations in these stromal components in both wild-type (WT) mice and a previously described model with altered Foxn1 expression (Foxn1lacZ/lacZ), which in itself offers an interesting framework for researchers in the field (Figures 1-2). The initial objective appeared to be exploring the role of Foxn1 and its targets in driving the thymic growth during early postnatal development.
The authors describe several alterations across the different epithelial, mesenchymal, and endothelial components, which become more pronounced and Foxn1-dependent as the thymus transitions into adulthood. Despite these later-life changes, the authors shift their attention to the first few weeks of postnatal life, noting that thymic growth during this early period (days 3, 7, and 14) describing to be Foxn1-independent, based on the seemingly identical profile of WT and Foxn1 mutant mice.
Exploring bioinformatic approaches to identify potential pathways involved in the early growth phase, the authors highlight IGF2 signaling as a possible candidate (Figure 3). Further analysis attempted to reveal the likely source of IGF2 as mesenchymal cells and its receptors located on thymic epithelial cells (TECs) (Figures 4-5), suggesting a potential IGF2-mediated mesenchymal-epithelial interaction. The manuscript concludes by demonstrating the expression of IGF2 in the human thymus (Figure 6).

© 2025. Published by The Company of Biologists under the terms of the Creative Commons Attribution License (https://creativecommons.org/licenses/by/4.0/).

While the scope of the manuscript is intriguing and the observations are of value, there are several aspects that should be addressed to strengthen the conclusions. These include further validation of the bioinformatic predictions, improved analysis of IGF2 in mesenchymal cells as well as additional functional assays to confirm the role of IGF2 signaling in TECs. Addressing these points would enhance the overall impact and robustness of the study's findings.

Specific points:
* 1. As a starting point, the authors should consider to include and/or discuss how the cellularity of TECs, endothelial, and mesenchymal cells evolve during the analyzed temporal window (d3-7-14-30). This would provide valuable insights into the expansion rates of these populations, offering a clearer understanding of the early thymic growth for the general reader.

Line graph showing TECs, endothelium, mesenchyme proportional change across time, separated by genotype added as a supplemental figure & briefly introduced on the results. While this is informative, it is relative change across time & is with a caveat.

* 2. The panels A-C in Figure 1 are complex to interpret at first glance. Given the clearer presentation and deconvolution of thymic stroma populations in panels D-G and Figure 2, it may be better to either move panels A-C to the supplementary section or omit them entirely. This would streamline the main figures and enhance the overall clarity of the manuscript.

We believe that it is quite important to see the overall data structure, which is pertinent to the story. As other reviewers did not have a similar suggestion, we kept these panels in the figure.

* 3. The evidence that Foxn1-dependent changes in TECs after day 14 lead to alterations in the diversity of TECs, mesenchymal, and endothelial cells (Figure 2 e.g. aaTEC, capillary ECs, mature medullary fibroblasts, in WT vs mut) is only superficially described and not thoroughly explored. While the review acknowledges that this may be the focus of another study, the authors should consider highlighting and discussing these alterations more explicitly.

A paragraph was added to highlight important changes in 14-day-old Z/Z.

MAIN: While the analysis suggests a potential involvement of IGF2, the conclusions, including the title, are overstated and require some attention. The data may suggest IGF2 as a possible factor, but further validation is needed to make definitive claims.

* 4a. Ligand enrichment analysis (Fig 3A). Do the authors also conduct similar analysis on day 3? Thymic expansion is a dynamic process. Is this purported signaling pathway already present on day 3?

Whilst CellChat can deduce intercellular interactions per sample basis, we conducted comparison analysis between two time points. We added another comparison between 3- and 7-day-old samples (separately for wild-type and Foxn1Z/Z) as a supplementary figure (S. Fig. 9). IGF2-IGF1R interactions are already present on day 3.

4b The analysis of IGF2 in immunofluorescence and the possible expression in mesenchymal cells (capsular fibroblast) is not very easy to appreciate and entirely convincing, mostly related with the co-localization of IGF2 staining and capsular fibroblasts (Fig5). The authors may attempt to better identify capsular and medullary fibroblast using other markers (e.g. gp38+ DPP4+) (Nitta et al., 2020).

We added SFig. 7 with GP38/Podoplanin-stained sections to further support decreasing capsular fibroblasts with age. The DPP4 antibody did not work in our fixed frozen samples.

* 4c. Can the authors provide functional evidence for a possible role of IGF2 in TEC? For example FTOCs/ or culturing ex vivo isolated TECs culture with recombinant IGF2. Assessment of proliferation, Foxn1 levels may be measured in these conditions.

© 2025. Published by The Company of Biologists under the terms of the Creative Commons Attribution License (https://creativecommons.org/licenses/by/4.0/).

In lieu of FTOC experiment, we provide functional evidence for the role of IGF signaling in the fetal/perinatal thymus using a TEC-specific conditional IGF1R knockout murine model (new Figure 6 & SFig. 7). Additional references were added to further support our hypothesis.

* 4d. The authors conclude from the results in Figure 6 that IGF2 expression increases in the human thymus. However, the results actually show a decrease in expression between the periods before and after 3 months, as well as between the thymus at 7 days old and at 13.5 months. Rather than an increase, we observe a decrease in expression between the younger and older thymus samples. The absence of an earlier time point, with lower expression of IGF2, prevents to claim that IGF2 increases in the neonatal thymus.

We rephrased this section to draw readers' attention to early IGF2 expression which drops after the transition point, consistent with the mouse results. We also added Figure 7N, an analysis of publicly available human scRNAseq datasets, which also supports this conclusion.

* 4e. Previous scRNA-seq studies of the human thymus have characterized distinct thymic stromal components (Park et al., Science 2020; Bautista et al., Nat Comm 2021). Could the authors analyze these available datasets to map IGF2 expression in human mesenchymal subsets and generate a similar representation to that shown in Figure 4 for the mouse datasets?

Bautista et al. (2021) had datasets most suitable to demonstrate our discovery (data density, data quality, annotation, etc.). We added Figure 7N with various non-TEC stromal subsets. The results are concordant with our discovery, but the temporal resolution is lacking.

Reviewer 3: SUMMARY OF THE ADVANCE MADE IN THIS PAPER AND ITS POTENTIAL SIGNIFICANCE TO THE FIELD

Overall, this is an interesting paper which sets up a mechanistic model to explain regulation of the transition from fetal growth to juvenile homeostasis during thymus development, that occurs at approximately P10 in mice. The authors first examine and appear to negate a smoking gun hypothesis, that this transition may be regulated by modulated Foxn1 expression levels. They then analyse transcriptional changes in thymic stromal cell types to identify IGF2 expression in thymic capsular fibroblasts as a strong candidate. Further analysis indicates tight regulation of IGF signalling in the developing thymus, with upregulation of the inhibitory receptor Igfr2 plus several Igfbps coinciding with the downregulation of Igf2 and the transition to homeostasis. Analysis of human datasets suggest conservation of this mechanism, corroborated by immune-staining. Finally, the authors present a model of how IGF2 expression in subcapsular and medullary fibroblasts, and arteriolar endothelial cells, likely regulates thymic epithelial cell (TEC)(and hence thymus) growth, including through stimulation of Cyclin D1 expression leading to RB hyperphosphorylation and thus both cell cycle progression and elevated Foxn1 expression. The manuscript is well written and the data well presented. The novel scRNAseq datasets included represent a substantial resource for the field, that will yield information beyond the focus of the current study. Functional testing of the IGF2 model is not presented, but some previous studies are relevant here and could be discussed - ameliorating this concern to some extent. Additionally, exploration of the mechanism leading to Igf2 (and Igfr1) downregulation would be welcome, as would be analysis of the effect of Foxn1 downregulation in non-TEC stroma the Foxn1Z mice (although this occurs after the transition under study it seems an omission to omit it here).

SUGGESTIONS TO AUTHORS
Issues that must be addressed
* There have been a number of previous studies describing effects of IGF2 overexpression on the thymus in addition to the Van der Ven paper already discussed (including the initial characterisation of the H2-Kb-IGF-II transgenic line). These papers provide further evidence relevant to the model presented and could usefully be considered in the discussion (e.g. Koojman et al PMID: 7751625 show effects on thymus cellularity consistent with the predictions of the current hypothesis, and van Buul-Offers et al PMID: 7537791 provide some preliminary findings on the effects of elevated IGF2 levels on thymus size). There is also some relevant early work from the Savino and Geenan (e.g. https://doi.org/10.1210/endo.141.3.7360) labs, that again could usefully

© 2025. Published by The Company of Biologists under the terms of the Creative Commons Attribution License (https://creativecommons.org/licenses/by/4.0/).

be considered in the discussion. The authors are requested to consider and discuss these papers (even briefly) in the context of the model they propose.

Thank you for the kind suggestions. Discussion was added with additional citations. Their results support our hypothesis, which certainly increases the impact of the manuscript.

* Related, the model proposed is complex, with effects of IGF2 likely on TEC and thymocytes. Are further clues (beyond changes in IGF pathway components) as to which compartment is primarily affected to cause the growth to homeostasis transition evident in the scRNAseq data (or are both equally affected), and as to the cause of IGF2 downregulation? Exploration and/or more extended discussion of this should be included.

We have added analysis of a TEC-specific IGF1R knock-out, which supports our conclusion that IGF2-mediated IGF signalling directly affects TECs. While we are obviously interested in what might be regulating this drop in IGF signaling, that is the focus of future study.

* Additionally, the data related to postnatal downregulation of Foxn1 in the Z/Z mice should be considered and presented in more depth. The authors currently deal with this in quite a cursory manner, noting that overall TEC phenotypes do not appear to have changed in the Z/Z mice at P14, while changes at P30 are consistent with a more recent report. However, in the original publication, Z/Z mice were shown to have smaller thymi by P14, presumably resulting from changes in the TEC compartment. Since the Z/Z mice appear from the original publication effectively not to go into homeostasis at this transition, further analysis of the Z/Z datasets to show the major impacts of reduced Foxn1 in the TEC compartment by P14 and on the non-TEC stroma at P14 and P30 should be presented. I appreciate that the authors may consider this separate to the focus of the paper, but since the scRNAseq are included in this study and the timeframe is related, it should be included here at least as supplementary information.

We added a paragraph to further highlight changes in 14-day-old Z/Z.

Minor issues
* Line 71 - define Foxn1 in the previous sentence.
Added a sentence how it is a transcription factor essential for thymic organogenesis.

* Line 88 - 'Instead' not correct here - instead of what?
'Instead' deleted. This was an editing error.

* Line 205 - good to add in a sentence reiterating that clear Foxn1-dependent changes were also observed but occurred after the P10 transition.
Added a sentence to also emphasise Foxn1-dependent changes post-transition.

* Fig 5 - Please show negative controls for IGF2 staining (as a supplementary figure), and comment on the source of the staining seen throughout the cortex. Is IGF2 expressed by thymocytes?
Added a supplemental figure, showing negative controls for murine IGF2, CD205, and APOD staining (w/ 7-day-old WT sample, which have plenty of IGF2 signal).
scRNAseq of murine peripheral T-cells do not show *Igf2* expression & there is no evidence IGF2 is expressed in thymocytes to our knowledge.
From our scRNAseq dataset, IGF2 staining seen throughout the cortex is likely endothelial cells/pericytes. Reflected in the results section.

* SF3 - Please show corresponding CD205 and DAPI stains.
SFig. 6 was added to show corresponding CD205 & DAPI stains.

Second decision letter

MS ID#: dev.204347R1

© 2025. Published by The Company of Biologists under the terms of the Creative Commons Attribution License (https://creativecommons.org/licenses/by/4.0/).

MS TITLE: Insulin-like growth factor 2 (IGF2) as a driving force of exponential expansion and differentiation of neonatal thymus

AUTHORS: Seung Woo Kang, Bryan R Helm, Yu Wang, Shiyun Xiao, Wen Zhang, Anusha Vasudev, Ken S Lau, Qi Liu, Ellen R Richie, Laura P Hale and Nancy Manley

Dear Nancy,
I am happy to tell you that your manuscript has been accepted for publication in Development, pending our standard publication integrity checks.

Reviewer 2: The authors have satisfactorily addressed this reviewer's concerns. The addition of new data using IGF1R conditional knockout in TECs provides a valuable enhancement that strengthens the study's conclusions.

Reviewer 3

SUMMARY OF THE ADVANCE MADE IN THIS PAPER AND ITS POTENTIAL SIGNIFICANCE TO THE FIELD

See initial review.

SUGGESTIONS TO AUTHORS

The authors have satisfactorily addressed all my concerns in their revised manuscript.

© 2025. Published by The Company of Biologists under the terms of the Creative Commons Attribution License (https://creativecommons.org/licenses/by/4.0/).
